# Lymphoid Hyperplasia and Lymphoma in Transgenic Mice Expressing the Small Non-Coding RNA, EBER1 of Epstein-Barr Virus

**DOI:** 10.1371/journal.pone.0009092

**Published:** 2010-02-08

**Authors:** Claire E. Repellin, Penelope M. Tsimbouri, Adrian W. Philbey, Joanna B. Wilson

**Affiliations:** 1 Division of Molecular and Cellular Biology, Faculty of Biomedical and Life Sciences, University of Glasgow, Glasgow, United Kingdom; 2 Division of Pathological Sciences, Institute of Comparative Medicine, University of Glasgow Veterinary School, Glasgow, United Kingdom; University of California San Francisco, United States of America

## Abstract

**Background:**

Non-coding RNAs have critical functions in diverse biological processes, particularly in gene regulation. Viruses, like their host cells, employ such functional RNAs and the human cancer associated Epstein-Barr virus (EBV) is no exception. Nearly all EBV associated tumours express the EBV small, non-coding RNAs (EBERs) 1 and 2, however their role in viral pathogenesis remains largely obscure.

**Methodology/Principal Findings:**

To investigate the action of EBER1 *in vivo*, we produced ten transgenic mouse lines expressing EBER1 in the lymphoid compartment using the mouse immunoglobulin heavy chain intronic enhancer Eμ. Mice of several of these EμEBER1 lines developed lymphoid hyperplasia which in some cases proceeded to B cell malignancy. The hallmark of the transgenic phenotype is enlargement of the spleen and mesenteric lymph nodes and in some cases enlargement of the thymus, liver and peripheral lymph nodes. The tumours were found to be of B cell origin and showed clonal IgH rearrangements. In order to explore if EBER1 would cooperate with c-Myc (deregulated in Burkitt's lymphoma) to accelerate lymphomagenesis, a cross-breeding study was undertaken with EμEBER1 and EμMyc mice. While no significant reduction in latency to lymphoma onset was observed in bi-transgenic mice, c-Myc induction was detected in some EμEBER1 single transgenic tumours, indicative of a functional cooperation.

**Conclusions/Significance:**

This study is the first to describe the *in vivo* expression of a polymerase III, non-coding viral gene and demonstrate its oncogenic potential. The data suggest that EBER1 plays an oncogenic role in EBV associated malignant disease.

## Introduction

Epstein-Barr virus (EBV) is a common human gamma-Herpesvirus infecting more than 90% of the world-wide population. It is normally contracted asymptomatically at an early age via saliva, persisting as a life-long infection in a latent state. If contracted post-puberty it can result in infectious mononucleosis, usually a self-limiting disease. However, EBV is also associated with several malignancies including Burkitt's lymphoma (BL), Hodgkin's disease and nasopharyngeal carcinoma [1]. In BL, EBV expression is restricted to the nuclear antigen 1 (EBNA1), the EBV encoded small RNAs (EBER1 and EBER2) and BART microRNAs (miRNAs) [2]; this limited expression pattern facilitates viral evasion of host immune defences. BL is also characterised by overexpression of c-Myc resulting from translocation of the gene to an immunoglobulin locus.

EBER1 and EBER2 (167 and 172 nucleotides, respectively) are RNA polymerase (pol) III transcripts and are highly conserved amongst EBV strains [3]–[5]. The secondary structures of the EBERs are predicted to include extensive double stranded regions with a number of short stem loops [3], [6]. These structures are conserved in the homologous *Papio* (baboon) Herpesvirus RNAs (HVP-1 and -2) [7], suggesting that they are critical for the EBERs interaction with specific proteins and their function. Several proteins bind to the EBERs, including double-stranded RNA-activated protein kinase (PKR), La antigen, ribosomal protein L22, 2′-5′ oligoadenylate synthetase, EBNA1 and retinoic acid-inducible gene I (RIG-I) [8]–[10].

The EBERs are hypothesised to disrupt the host cell interferon response, but this role is currently ambiguous. They can stimulate type I interferon (IFN) through recognition by the dsRNA binding protein RIG-I [10]. Conversely, EBER1 was shown to inhibit IFNα induced apoptosis and a suggested mechanism for this is through binding to PKR thereby inhibiting PKR auto-phosphorylation and thus blockade of downstream events [11], however, this proposed mechanism has been questioned [12]. Ruf *et al*. observed no differences in the levels of phosphorylated PKR or its substrate following IFNα treatment of EBV positive and negative cells, leaving the mechanism by which the EBERs mediate resistance to IFNα-induced apoptosis currently unresolved. These observations are further complicated by the finding that in trafficking assays EBER1 appears to be confined to the nucleus [13] while PKR and RIG-I are largely cytosolic.

The EBERs are expressed in virtually all EBV associated tumours and are the most abundant viral transcripts in several latently infected B-cell lines [14]. Their ready detection allows them to be used as indicators for the presence of EBV in infected cells by *in situ* hybridisation [15]. A potential role of the EBERs in malignant pathogenesis was suggested by the observation that they can render lymphoma cells more malignant and protect them from some apoptosis inducing agents, such as IFNα [16]-[19]. Furthermore, the EBERs promote the induction of autocrine growth factors interleukin (IL)-10, IL-9 and insulin-like growth factor-1 [20]–[22]. A functional difference between the EBERs has been described, where EBER2 but not EBER1 was found to promote EBV mediated B-cell immortalisation *in vitro*, at least in part through IL-6 induction [23] and an EBER2 specific analysis of changes in cellular expression has been conducted [24]. However, for most studies the role of the two EBERs has not been separated and thus their individual functions remain elusive. In addition, prior to the study described here, there has been no animal model for analysing the function and mechanisms of the EBERs *in vivo*.

To investigate the independent role of EBER1, we have generated transgenic mice expressing EBER1 in the lymphoid compartment using its viral pol II/pol III promoter and included a cellular lymphocyte specific pol II enhancer. Several lines of EBER1 mice developed lymphoid hyperplasia and in some cases B cell lymphoma. In order to model the situation in BL, EBER1 expressing mice were then cross-bred with transgenic lines expressing either c-Myc or EBNA1, or N-Myc as a further comparison, and latency to lymphoma was examined.

## Results

### Generation of EBER1 Transgenic Mice

**Figure 1:**
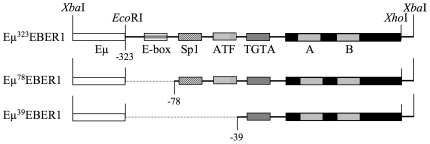
The EνEBER1 transgenes. The Eμ cassette (white box) was placed 5′ of three EBER1 *Eco*RI-*Xho*I gene fragments which include promoter sequences from −323, −78 or −39 from the canonical start of EBER1 transcription. EBER1 promoter motifs: E-box, Sp1 and ATF binding sites and TGTA box are indicated. All constructs include the EBER1 transcription unit (black) with pol III “A” and “B” boxes.

To explore a potential EBER1 induced lymphoid phenotype *in vivo*, transgenic mice were generated designed to express EBER1 in the lymphoid compartment. The natural viral transcriptional control unit of EBER1 is composed of a hybrid of classical RNA pol II and pol III motifs. The 5′ promoter incorporates two pol II motifs (Sp1 and ATF binding sites) upstream of a TATA-like box (TGTA). Further upstream, two E-boxes were identified and provide c-Myc binding sites [25]. Within the transcription unit are “A” and “B” boxes typical of type II pol III promoters [26]. In order to attempt to restrict expression to the lymphoid compartment, the pol II murine immunoglobulin heavy chain (IgH) intronic enhancer (Eμ) was placed at the 5′ end of three constructs, with progressive deletion of the EBER1 promoter (Figure 1). The competence to express EBER1 from the three constructs was confirmed by quantitative (q)RT-PCR and northern blotting following transient transfection into a murine B-cell line (data not shown).

Nineteen transgenic mouse founders were generated with the EμEBER1 constructs, from which 13 lines were established (supplementary Table S1). The integrated transgene head-to-tail tandem copy number was estimated by slot blot from 1^st^ generation offspring and ranged from 2 to approximately 200 (supplementary Table S1). Lines 127 (Eμ^78^EBER1) and 138 (Eμ^39^EBER1) demonstrated an X-linked pattern of inheritance with all others being autosomal (not shown).

**Figure 2:**
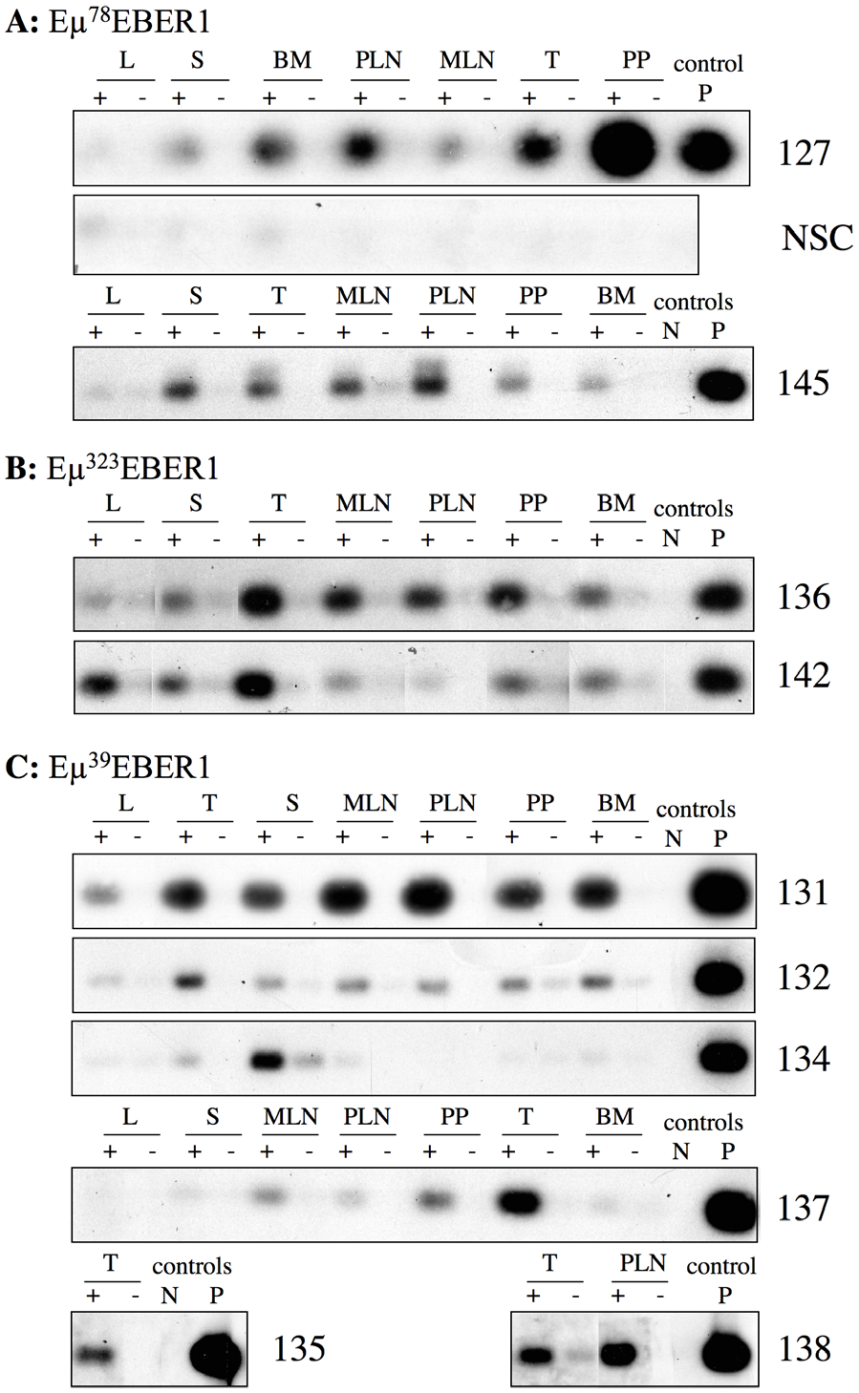
EBER1 expression pattern in mouse lymphoid tissues. Transgene expression was examined in mice of Eμ^78^EBER1 lines and a transgene-negative sibling control (NSC) (**A**), Eμ^323^EBER1 lines (**B**) and Eμ^39^EBER1 lines (**C**) and detected using RNA derived from tissues of 2 to 4 month old mice by RT-PCR using a gene specific RT primer (CR4). PCR (CR8/CR9) products were Southern blotted and hybridised with an EBER1 probe. Data for lymphoid tissues are shown, including spleen (S), thymus (T), bone marrow (BM), peripheral lymph nodes (PLN), mesenteric lymph nodes (MLN), Peyer's patches (PP) and liver (L). For each RT (+) reaction a no RT (−) control was included. PCR controls included a water only negative (N) control and a plasmid DNA amplified positive (P) control. Quality and quantity of all RNAs analysed was assessed and controlled for by RT-PCR for GAPDH, using oligodT for the RT reaction and GAPDH specific primers.

**Figure 3:**
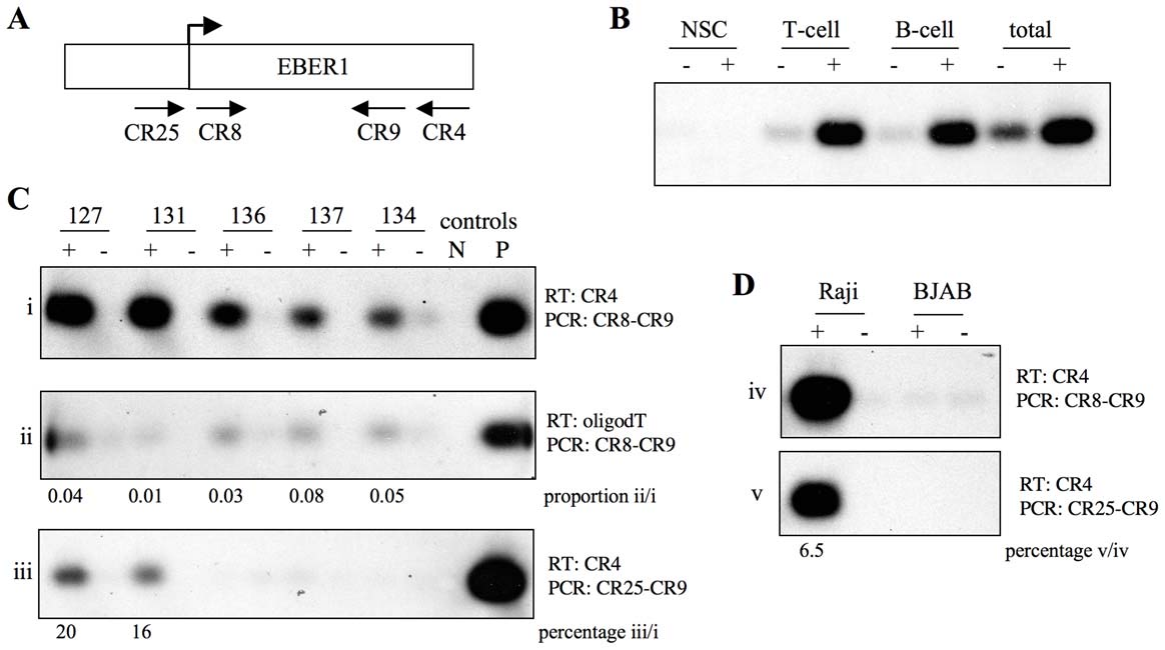
Analysis of the transgenic EBER1 transcripts. (**A**) Schematic diagram of the EBER1 gene showing the canonical start of transcription (arrow) in relation to the primers used. (**B**): Cells isolated from Peyer's patches collected from 3 (pooled) line 127 mice were separated into B and T-cell fractions and RNA prepared (along with a pre-selection aliquot “total” and Peyer's patch sample from 3 pooled NSC). EBER1 expression was assessed by RT-PCR using gene specific RT primer (CR4) and CR8/CR9 PCR. PCR products were Southern blotted and hybridised with an EBER1 probe. (**C** and **D**): RNA from lines 127 (Peyer's patches), 131 (peripheral lymph nodes), 136 (thymus), 137 (thymus), 134 (spleen) and BL cell lines Raji and BJAB (EBV positive and negative respectively), was assessed by RT-PCR. The RT reaction was performed using an EBER1 specific primer (CR4) (panels i, iii, iv and v) or an oligodT primer (panel ii). The RT reaction was followed by PCR using CR8-CR9 primers (panels i, ii and iv) or CR25-CR9 (panels iii and v). The products were Southern blotted and hybridised with an EBER1 probe. For each RT (+) reaction a no RT (−) control was included. Three controls were performed for the PCR, a negative (N) water only control and plasmid DNA amplified positive (P) control and routinely oligodT-GAPDH (not shown) for quality control. The membranes shown in panels i, iii and iv were exposed for 16 hours, whereas ii and v were exposed for 3 days. Relative signal intensity is given for samples in panels ii and iii compared to i and for panel v compared to iv.

Ten out of eleven lines assessed for transgene expression showed EBER1 expression in at least one lymphoid tissue (Figure 2). The expression pattern in the lymphoid tissues differs between lines; for instance line 127 expresses EBER1 to varying degrees in the lymphoid tissues with Peyer's patches (PP) showing highest expression, whereas line 131 shows relatively equal expression levels across the lymphoid tissues. A comparative expression analysis (by quantitative RT-PCR) was conducted between samples from different lines and compared to the Akata EBV positive B-cell line (supplementary Figure S1). This showed that for Peyer's patches samples the levels of EBER1 expression (highest first) was lines 127, then 131, 142, 137, 136 and for thymus the order was 142, 127, 131, 137, 136. The level of EBER1 transcript detected in the tissues was three orders of magnitude lower than that quantified in the clonal Akata BL cell line. Cells of the spleen and Peyer's patches of the highest expressing line (line 127) were separated into B-cell and T-cell fractions, revealing EBER1 expression in both populations (shown for Peyer's patches, Figure 3B). However, line 134 shows almost exclusive expression to the spleen and 137 to the thymus (Figure 2) which would suggest B-cell and T-cell specific expression in these two lines (respectively). Expression in non-lymphoid tissues was also examined in lines 127, 131, 136 and 137 (supplementary Figure S2). Line 127 showed no expression in other tissues with the exception of the brain, whereas line 131 showed expression in several non-lymphoid tissues.

The expression patterns and levels show no correlation with the transgene copy number (as is usual), or with the three different constructs used and are likely to be influenced by the chromatin configuration at the sites of transgene insertion. Efficient transcription of the EBER genes has previously been shown to require the native upstream pol II elements (ATF, Sp1 and E-boxes) [5]. These elements have been completely deleted from the Eμ^39^EBER1 construct used here, which nevertheless shows expression at comparable levels to the transgenes retaining these elements (Eμ^78^EBER1 and Eμ^323^EBER1) (Figure 2 and supplementary Figure S1). Therefore deletion of the native EBER1 promoter pol II elements appears to have been compensated for by the inclusion of the Eμ element (which incorporates E-boxes). Lymphoid restricted expression was achieved in some lines, notably in lines 134 and 137 with the Eμ^39^EBER1 transgene, suggesting that deletion of the EBER1 Sp1 and ATF elements may have achieved this restriction in some cases.

### EBER1 Is Probably Transcribed by Pol III in the Transgenic Tissues

In order to explore if transgenic EBER1 is transcribed by pol II or pol III, reverse transcriptase (RT) reactions were performed using the highest expressing tissues of lines 127, 131, 134, 136 and 137, with either an EBER1 specific RT primer CR4 (Figures 3A and 3C, panel i) or an oligodT primer to copy pol II mRNA sequences (Figure 3C, panel ii). The RT products were amplified by PCR using EBER1 specific primers (CR8/CR9) and the products quantified (Figure 3C panel ii). Expression was readily detected using the EBER1 specific RT primer CR4 (Figure 3C, panel i) with faint signal detection (0.01 to 0.08 fold compared to CR4) using the oligodT RT-PCR (Figure 3C, panel ii). These results indicate that the majority (if not all) of the EBER1 transcripts in these tissues are not polyadenylated and therefore are unlikely to be transcribed by pol II using a cryptic or fortuitous cellular polyA addition signal.

An RNA start upstream of the canonical EBER1 start is used to a minor extent in the transgenic lines 127 and 131 (20% or less) as was described for the EBV positive BL cell line Raji [27], [28] (Figure 3C, panel iii and 3D, panel v). It was detected using an upstream PCR primer (CR25) following gene specific RT. The biological significance of this is unknown, but the longer transcript could fold differently with functional consequence.

### EBER1 Expression Does Not Affect B and T Cell Proportions in the Lymphoid Tissues of Young Transgenic Mice

We sought to determine if EBER1 expression *in vivo* affects either B and T cell proportions or their differentiation status in the lymphoid tissues of these mice, prior to the development of any phenotype. Tissues of young mice of lines 127 and 131 were examined by flow cytometry. B cells were assessed with B220 and T cells with CD3 and Thy1.2 markers. No differences were observed in the proportions of B and T cells in the lymphoid tissues of lines 127 and 131 (supplementary Figure S3). Lymphoid expression of several markers for differentiation status were examined (supplementary Table S5), for line 131 no differences from controls were observed for any of the lymphoid tissues and antibodies tested (data not shown), suggesting that for this line the status of the B and T cells (with respect to the antibodies used) is not modified by the presence of the transgene. For mice of line 127, the only difference observed between transgenic and transgene-negative sibling control (NSC) tissues was a possible perturbation of the longer lived B1a B-cell sub-population in the Peyer's patches, the highest expressing tissue of this line (supplementary Figure S3, panel E). A greater proportion of the B1a B-cell sub-population in the Peyer's patches was observed in the transgenic mice compared to NSC.

### The Levels of PKR and eIF2α Phosphorylation Are Unchanged in EBER1 Transgenic Mice

**Figure 4:**
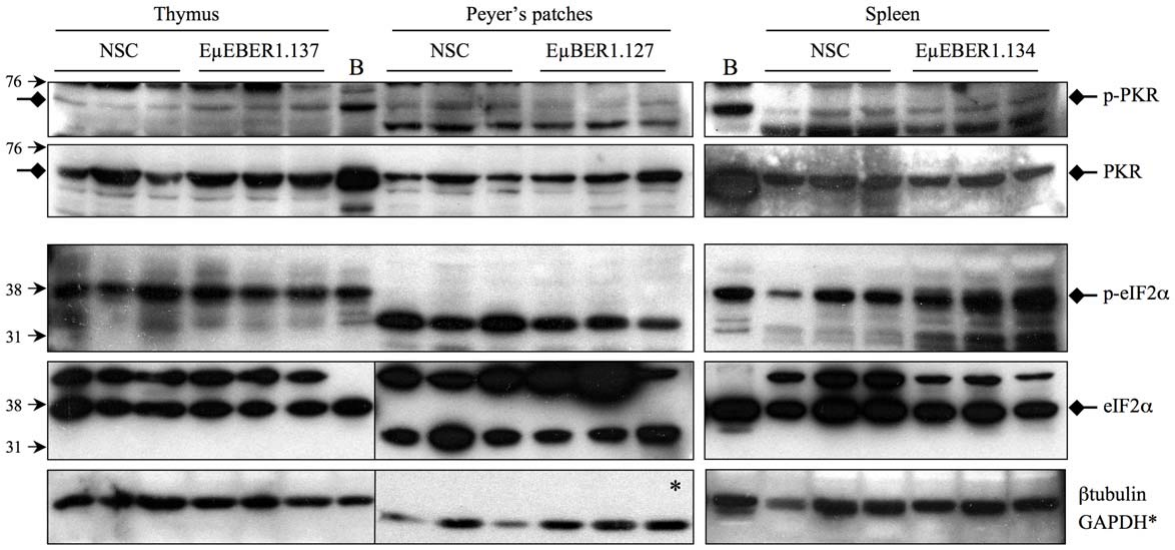
Steady state phosphorylated and total PKR and eIF2α levels in the EBER1 mice. Steady state PKR (68 kD) and eIF2α (40 kD) levels, along with their activated phosphorylated forms (as indicated) were assessed by western blotting (for PKR: 7.5% SDS-PAGE, for eIF2α: (10% SDS-PAGE). Protein extracts from three biological replicates from the highest expressing tissues from each of three lines were examined: thymus from EμEBER1 line 137, Peyer's patches from EμEBER1 line 127 and spleen from EμEBER1 line 134 along with NSC replicates for each tissue. The human BL cell line BJAB (B) treated with IFNα has been used as a positive control. Detected protein levels were quantified and normalised against β-tubulin (55 kD) levels or for Peyer's patches* against GAPDH (37 kD) levels. Note: a 40 kD band for eIF2α could not be detected in Peyer's patch tissues (even after long exposure as shown in the inset panel for eIF2α Peyer's patch samples), however the α-eIF2α antisera did react with a band of approximately 33 kD which was also detected by the α-phospho-eIF2α antisera (the former only, also reacting with a murine band at 43 kD), which could be a tissue specific derivative of eIF2α. Molecular weight markers are shown to the left.

EBER1 has been shown to inhibit PKR autophosphorylation in cell free systems [29], however EBER1 shows PKR independent action in cultured cells [12], [30]. To explore if EBER1 expression in the transgenic mice impacts PKR, steady state levels of PKR and one of its substrates, the alpha subunit of the eukaryotic initiation factor 2 (eIF2α) and the activated, phosphorylated forms of both, were examined in three of the transgenic lines in adult mice prior to the development of any phenotype. The highest EBER1 expressing tissue from the three mouse lines; 127, 134 and 137, namely Peyer's patches, spleen and thymus, respectively were assayed compared to NSC tissues. Three biological replicates were examined in each case and no reduction in steady state or phosphorylated levels of PKR were observed in the EBER1 tissue compared with NSC (Figure 4). Similarly no reduction in level or phosphorylation state of eIF2α was observed for the thymus of 137 and the Peyer's patches of 127 samples (Figure 4). For line 134 spleen samples, an apparent reduction in the levels of total eIF2α was detected in the transgenic samples compared to NSC, but no reduction in phosphorylated eIF2α levels (as a PKR target) was observed. Splenic samples of line 127 mice did not show this reduction in total eIF2α levels (not shown) and thus further characterisation of this observation is required.

### EBER1 Transgenic Mice Develop Lymphoid Hyperplasia and B-Cell Lymphoma

The phenotype of the EBER1 transgenic mice was monitored alongside NSC. Mice of several lines displayed a pronounced preneoplastic enlargement of lymphoid tissues, particularly splenomegaly with lymphoproliferation, also noted in the thymus, peripheral lymph nodes and Peyer's patches (Table 1). Lymphoma was observed in mice of all the lines and the incidence has been compared to spontaneous lymphoma in NSC (Table 1). A proportion of NSC mice typically show lymphoid expansion or develop lymphoma, particularly in the age group over 20 months. Nevertheless, the incidence of lymphoid expansion or lymphoma was significantly higher for lines 127, 131, 134 and 135 compared to NSC.

**Table 1:** Summary of the lymphoid pathology in EμEBER1 lines.

Tg Line Age (mo)	127	131	132	134	135	136	137	138	142	145	negative sibling control
>19 – ≤24	16 (**13**)/24	7 (**4**)/11	1 (**1**)/6	7 (**3**)/9	4 (**2**)/4	4(**2**)/8	3 (**2**)/10	1 (**1**)/4	5 (**1**)/9	6 (**4**)/13	30 (**6**)/122
>14 - ≤19	7 (**7**)/8	½			3/4		½	1/2	0/1	0/3	3 (**3**)/24
>10 – ≤14	3 (**2**)/5	1 (**1**)/4	2/3	0/4		0/3	0/3	3/5		0/4	3 (**2**)/33
>6 – ≤10	14 (**3**)/18	0/5	0/3	0/9	1/1	5/16	14 (**2**)/21	1/12	0/7	2/8	2/66
>3 – ≤6	2/15	0/13	0/2	2/2	0/1	0/3	5/12	0/8	1/2	0/20	0/151
≤ 3	4/6	0/2		0/1	0/1	3/3		0/1		0/3	0/51
Total	46/76	9/37	3/14	9/25	8/11	12/33	22/48	6/32	6/19	8/51	38/447
Percentage	60.5	24.3	21.4	36	72.7	36.4	45.8	18.8	31.6	15.7	8.5
P χ^2^	<0.001	<0.025	<0.1	<0.001	<0.001	<0.001	<0.001	<0.0525	<0.001	<0.1	
**Analysis of** >19 – ≤24	16 (**13**)/24	7 (**4**)/11	1 (**1**)/6	7 (**3**)/9	4 (**2**)/4	4 (**2**)/8	3 (**2**)/10	1 (**1**)/4	5 (**1**)/9	6 (**4**)/13	30 (**6**)/122
Percentage	66.7	63.6	16.7	77.8	100	50	30	25	55.6	46.2	24.6
P χ^2^	<0.001	<0.001	<0.9	<0.001	<0.001	<0.05	<0.5	<0.8	<0.02	<0.05	
**Analysis of** >10 - ≤24	26 (**22**)/37	9 (**5**)/17	3 (**1**)/9	7 (**4**)/13	7 (**2**)/8	4 (**2**)/11	4 (**2**)/15	5 (**1**)/11	5 (**1**)/10	6 (**4**)/20	36 (**11**)/179
Percentage	70.3	52.9	33.3	53.8	87.5	36.4	26.7	45.5	50	30	20.1
P χ^2^	<0.001	<0.01	<0.8	<0.01	<0.001	<0.2	<0.9	<0.05	<0.05	<0.3	

The number of transgenic positive animals which have either developed lymphoma or shown signs of lymphoid expansion, out of the total number of animals examined in the different age categories are shown (number with lymphoid pathology (bold: of which were neoplastic)/total number of mice in the category). The majority of negative mice examined in the 19-24 months category were taken at 23/24 months at the end of study. Generally mice examined under 1 year old were taken in cohort groups (without phenotypic selection) for particular studies (eg. expression analysis). The percentage of mice with tumour-expansion compared to no tumour is indicated for each line. The P values were calculated from a chi squared (P χ^2^) test. To avoid bias due to the large number of negative sibling control mice taken under the age 10 months, largely phenotype free, the percentage of mice with lymphoid expansion and chi squared tests are also shown for the older age groups alone (>10≤24 months and >19≤24 months, in the panels below).

**Figure 5:**
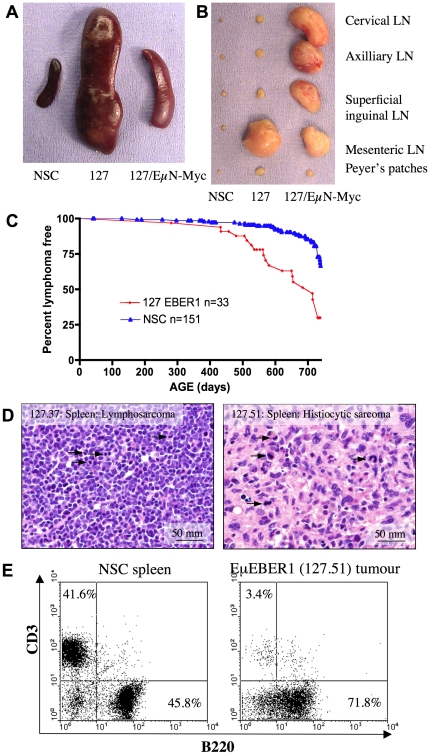
The lymphoma phenotype of Eμ^78^EBER1 line 127 mice. The lymphoma phenotype observed in a transgenic Eμ^78^EBER1 line 127 mouse (127) and a 127xEμNmyc bitransgenic mouse compared to a NSC mouse, seen in the spleen (**A**) and lymph nodes (LN) (**B**). The 127 transgenic mesenteric lymph node shown was a portion of a large interconnected series of nodes. (**C**) Kaplan Meier plot of line 127 Eμ^78^EBER1 transgenic mice (127 EBER1) (red, n = 33) and NSC (blue, n = 151) mice comparing lymphoma incidence, log rank t test p<0.0001. The study end point was 2 years. (**D**) Photomicrographs of H&E stained sections of Eμ^78^EBER1 line 127 tumours taken at original magnification 400x (scale bar shown). Several mitotic Figures are labelled with arrows. Note: lymphosarcoma 127.37 revealed IgH gene rearrangements (supplementary information) and histiocytic sarcoma 127.51 showed B220 staining (seen in panel E), but no detectable IgH rearrangements. (**E**) Flow cytometry of a 127 splenic tumour (right) and NSC spleen (left) staining with the T-cell and B-cell specific markers, CD3 and B220 respectively. B- and T-cell quadrant percentages are shown.

Subsequent studies of EBER1 transgenic mice were focused on line 127, as the highest expresser. For line 127, a cohort study over 2 years has been completed, in which 58% (19/33) of the transgenic mice succumbed to lymphoproliferative disease characterised by extensive pre-neoplastic lymphoid expansion or lymphoma ranging from 14 to 24 months of age, compared to 23% (35/151) of NSC mice which ranged from 18 and 24 months (Figure 5). The difference in frequency of this phenotype between line 127 transgenic and NSC mice is statistically significant (P<0.0001). The transgenic tumour phenotype typically presented as a massive enlargement of the spleen and mesenteric lymph nodes, some expansion of the thymus, with extensive invasion of the liver in some cases and occasional involvement of the peripheral lymph nodes and Peyer's patches (Figure 5A and B). The tumour pathology was noted as either lymphosarcoma (lymphoma) or neoplastic cells resembling histiocytes (histiocytic sarcoma) (Figure 5D). Splenic tumours of mice of line 127 analysed by flow cytometry were found to be of B-cell origin, staining positive for the B-cell specific surface marker B220 and negative for the T-cell surface marker CD3 (Figure 5E). In addition, some tumours showed clonal IgH gene rearrangements (supplementary Figure S4).

IL-10 secretion was examined by ELISA but not detected in serum samples from five line 127 tumour bearing mice or five NSC serum samples (all samples showing no IL-10 compared to a positive standard curve, thus there is no graphical data to show). Analysis by cytokine array showed a negligible signal for IL-10 and IL-9 in a pool of three splenic tumour tissue samples, which was equivalent to NSC splenic samples. Therefore there is no evidence to suggest that EBER1 is acting to induce levels of IL-10, either in the serum or tumours in these mice.

### Does EBER1 Cooperate with Myc in Lymphomagenesis *In Vivo*

**Figure 6:**
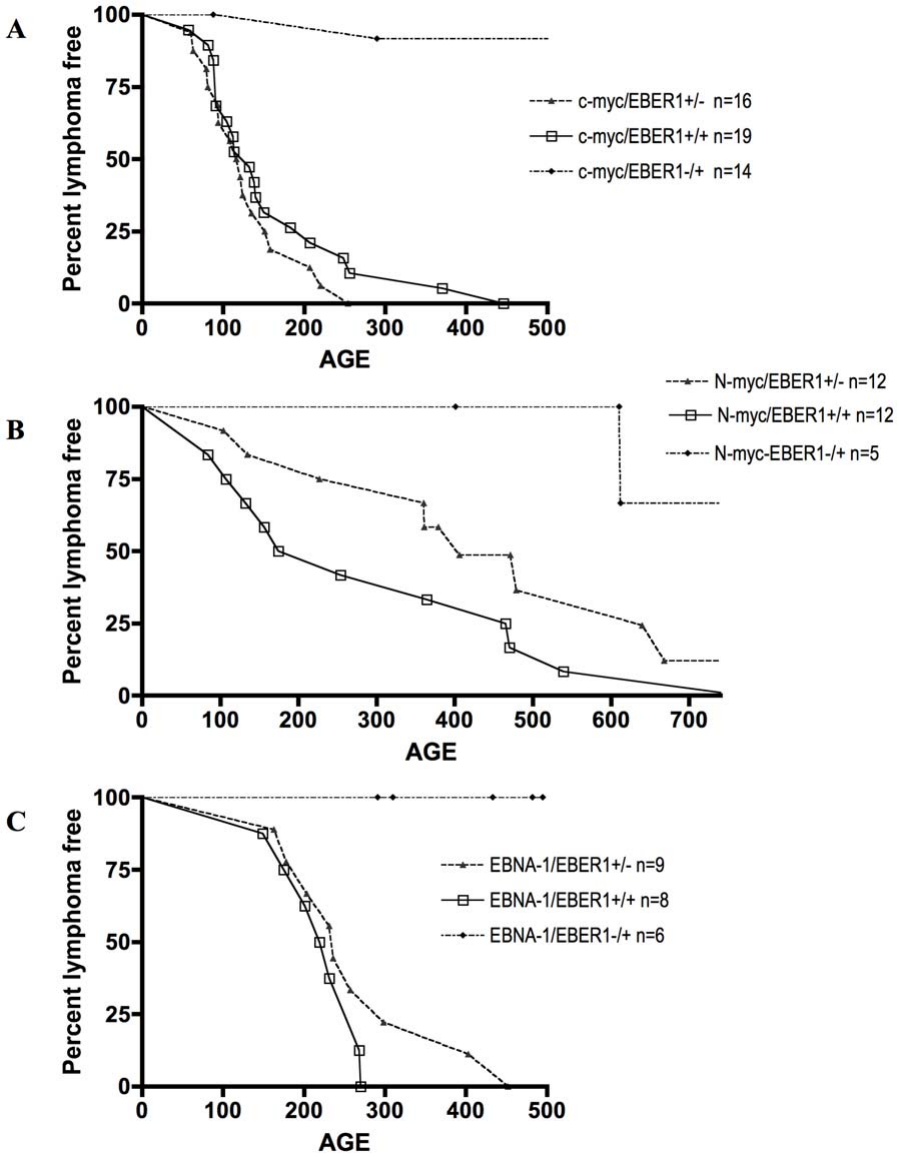
Latency to lymphoma in EBER1 mice compared to EBER1/Myc and EBER1/EBNA-1 bitransgenic mice. Kaplan Meier plots for lymphoma incidence from cross breeds of EμEBER1.127 with Eμc-myc (**A**), or EμN-myc (**B**) or EμEBNA-1 (of the line 26) (**C**) mice are shown. Bitransgenic mice in each case are shown with the open box symbol. (**A**) Study end point: 500 days, log rank t test P = 0.2803. (**B**) Study end point: 740 days, log rank t test P = 0.1308. (**C**) Study end point: 500 days, log rank t test P = 0.263. These data show no significant difference in the lymphoma incidence curves between EμMyc or EμEBNA-1 single transgenic compared with the relevant bitransgenic mice.

In oncogene-carrying transgenic cross breeds, reduced tumour latency in bitransgenic animals can indicate that the two oncogenes act with functionally different consequences, while no cooperation can (but not necessarily) suggest that the two oncogenes may employ functionally redundant mechanisms, even if the mode of action is highly divergent. In order to explore this with the tumour prone EBER1 line 127, the mice were cross bred with the well characterised Eμc-myc and EμN-myc lines of mice [31], [32]. Tumours arising in the bitransgenic mice phenotypically resembled those arising in the Myc mice with extensive involvement of the lymph nodes (Figure 5A and B). No acceleration to tumour onset was observed between EμEBER1.127 and Eμc-myc mice (Figure 6A). While bitransgenic mice in the EμEBER1.127 and EμN-myc cross on average showed a reduced latency to tumour onset compared to EμN-myc mice (median of 214 days compared to 406 days, Figure 6B), the survival curves were not found to be statistically significantly different. While these data do not demonstrate cooperative action between Myc and EBER1 in lymphomagenesis, they do not disprove the possibility. Similarly, no cooperative action in tumour latency between EBER1 and the viral nuclear antigen EBNA-1 was revealed by this approach (Figure 6C).

**Figure 7:**
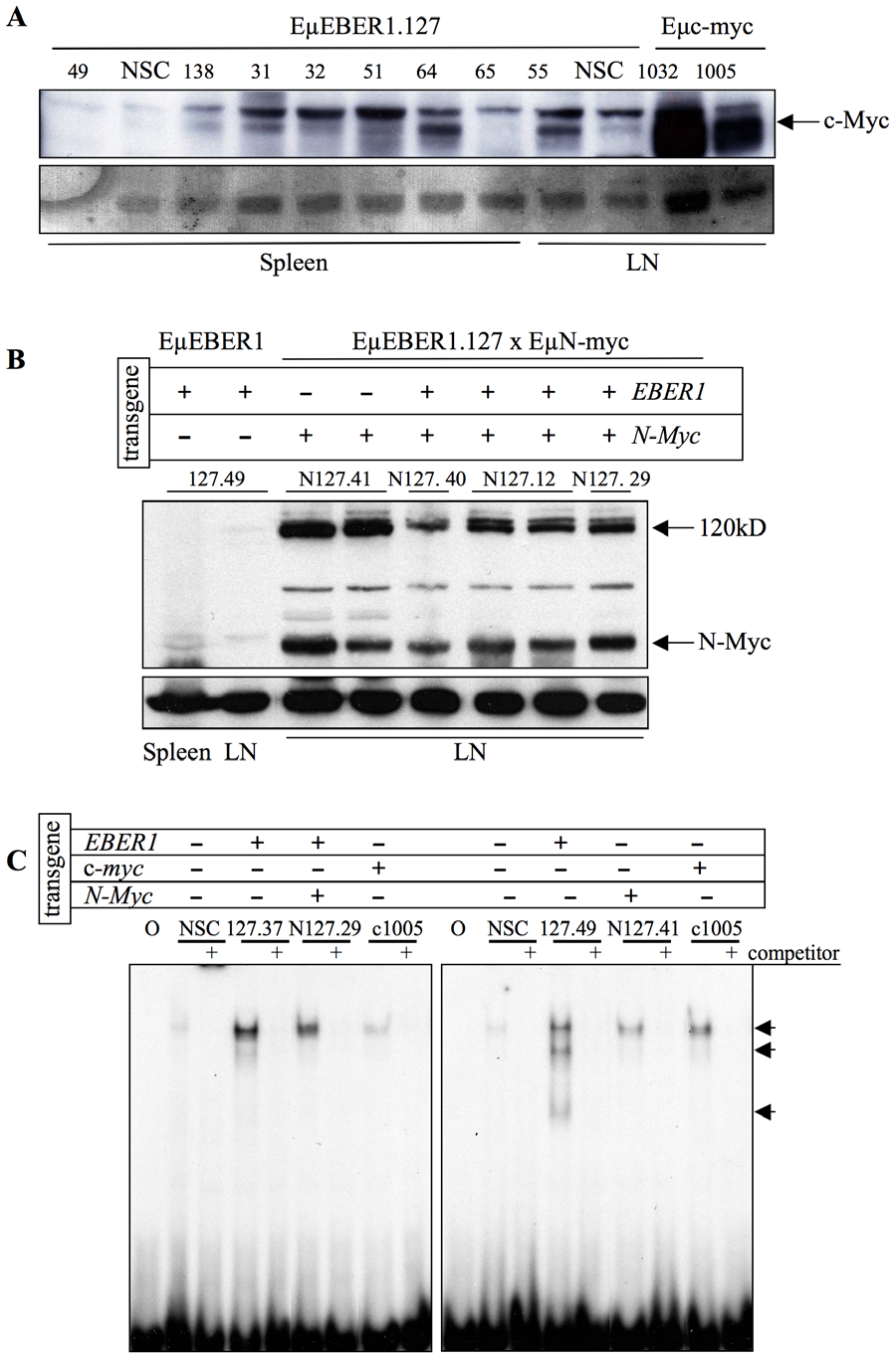
Analysis of Myc expression and DNA binding activity in transgenic lymphomas. (**A** and **B**) Western blots of 100µg protein extracts from tumours samples from spleen or lymph node (LN) tissues, from EμEBER1 line127, or Eμc-myc or EμEBER line 127 crossed with EμN-myc mice are shown, probed with antibodies to c-Myc, 67 kD (**A**) or N-myc, 67 kD (also detecting another antibody specific band at 120 kD) (**B**). Sample loading was assessed by Ponceau staining of the blot (lower panel A) or from non-specific band staining (lower panel B). (**C**) EMSA for E-box binding activity of nuclear extracts of tumour samples from mice of EμEBER1 line 127 (127.37 and 127.49), a bi-transgenic EμEBER1/EμN-Myc (N127.29), an EμN-Myc (N127.41) and Eμc-myc (c1005) and non tumour NSC (as indicated above the tracks. All samples were assayed with an E-box oligo and a track with free oligo/probe is indicated by “O”. The addition of 100 x unlabelled competitor to a sample is indicated by (+). The specific bands are indicated.

In order to explore this further, Myc expression and activity in the EBER1 tumours was examined. Again, if EBER1 function was redundant with Myc, one would not expect induction of Myc to serve any selective advantage in the process of tumourigenesis and therefore would not expect to detect induced expression in the ensuing tumour. Alternatively, if EBER1 acted through induction of Myc, constitutive Myc expression would be observed. Induction of c-Myc expression was observed in several, but not all, of the EμEBER1.127 lymphomas (Figure 7). Furthermore, activation of E-box DNA binding (to which Myc family proteins bind) was also observed in EBER1 tumour samples (Figure 7C), even in the absence of detection of c-Myc or N-Myc (Figure 7, note tumour sample 127.49). These data indicate that the function of EBER1 in tumourigenesis is neither redundant with Myc nor acts through induction of Myc, but may be cooperative.

## Discussion

This report describes expression of a pol III transcribed transgene displayed by several lines of mice which, in some lines, is restricted or biased to the lymphoid compartment. Despite the low level of EBER1 expression achieved in these transgenic mice a phenotype of lymphoid organ enlargement, notably splenomegaly, was evident in several independent lines with progression to late onset lymphoma, particularly apparent in line 127 with highest EBER1 expression. These data provide the first evidence that a pol III non-coding RNA has oncogenic potential *in vivo* and could implicate EBER1 in the initiation or progression of EBV associated malignant disease.

To explore the mode of action of EBER1 as an oncogene, cross-breeding studies were undertaken. EBER1 and EBNA1 do not appear to cooperate in reducing the latency to lymphomagenesis in this system, nor do EBER1 and Myc. Thus the function of EBER1 as a predisposing factor in tumourigenesis cannot be classified by this approach. However, c-Myc induced expression and DNA binding activity was detected in several EBER1 tumours. This does not imply that EBER1 induces Myc, indeed the differing levels of Myc in the tumour samples (in some cases Myc is not detected) would argue against this. Instead this suggests that Myc has been induced or activated in some instances (for example by mutation or epigenetic modification) during the progressive steps to tumourigenesis and has thereby contributed to the development of the lymphoma. This function would not be selected for in tumour development if the action of Myc was redundant with that of EBER1 (the latter activity already being present in the EBER1 expressing tissues) and as such the data indicate that Myc and EBER1 action are not redundant in tumourigenesis and potentially could cooperate in ways not assessed by the tumour latency assay described.

In BL cell lines EBER expression has been shown to lead to increased expression of the immunosuppressive cytokine IL10 [20]. Thus EBER1 might play a role in permitting the survival of an infected cell through disruption of the immune system. This is supported by the observation that EBER1 inhibits IFN-α induced apoptosis, which has been proposed (though controversial) to act via the binding and inhibition of PKR auto-phosphorylation [11], [12]. PKR, which inhibits protein synthesis through the phosphorylation of eIF2α, has been suggested to have tumour suppressor properties since a functionally defective PKR has been shown to enhance transformation and tumourigenesis of NIH3T3 cells [33], [34]. In our studies, we could not detect any induction of IL-10 or inhibition by EBER1 of steady state levels of phosphorylated PKR or its substrate eIF2α *in vivo*, as was also noted by Ruf *et al.* in BL cells [12]. However, it remains possible that EBER1 expression could influence IL-10 levels or PKR activation following immune challenge. Furthermore, EBER1 expression in these mice is low and very high levels of EBER1 may be required to detect disruption of PKR activity. Nevertheless, this raises the possibility that EBER1 is not acting through these routes to induce the observed phenotype.

EBER1 might regulate cell survival or growth through modulation or sequestration of its protein partners. Alternatively it might directly regulate gene expression as a small RNA. Small non-coding RNAs, particularly miRNAs represent a class of post-transcriptional regulatory molecules which have diverse functions, including the control of cell growth [35]. Indeed miR155 has been shown to lead to lymphoma in transgenic mice [36] and the EBV encoded miRNA BHRF1-3 appears to suppress an anti-tumour, T-cell-attracting chemokine, CXCL-11 [2]. Twenty two miRNAs expressed by EBV have been identified, none of which incorporate EBER sequences [37]–[39]. Moreover, there is no evidence to indicate that EBER1 is processed by Dicer [40] to form a miRNA and it does not bind to exportin 5 [13]. However, it cannot be ruled out that EBER1 might be processed and act in an as yet undefined way.

While EBER1 levels are often very high in BL cell lines and EBV positive lymphoblastoid cell lines, the relative levels can vary enormously, for example EBER1 levels are 100 fold higher in the BL cell line Raji compared to Akata and it is not clear how this compares to the levels that were expressed in the primary tumour cells. The expression level achieved in the transgenic mice in this study is low, nevertheless it is sufficient to have induced an altered phenotype. As such, these mice provide a means to study the mechanism of action of this small RNA *in vivo*, both its impact upon immune function and its role in tumourigenesis.

## Materials and Methods

### Ethics Statement

All procedures were conducted according to UKCCCR Guidelines for the Welfare of Animals in Experimental Neoplasia and under UK Home Office license and the research has complied with Home Office and institutional guidelines and policies.

### Construction of Plasmids

EBER1 plasmid pLEXIII [30] was used as a PCR template to generate ^323^EBER1, ^78^EBER1 and ^39^EBER1 (differing in promoter length) using primers CR1, CR2, or CR3 forward primers with CR4 reverse primer (all primer sequences can be found in supplementary Tables S2 and S3), 30 cycles, annealing at 60°C. The *Eco*RI-*Xho*I digested PCR products were ligated to pcDNA-Eμ vector (Eμ is the murine immunoglobulin heavy chain (IgH) intronic enhancer and its sequence was derived from pEμLMP1 [41]) to generate pEμ^323^EBER1, pEμ^78^EBER1 and pEμ^39^EBER1 plasmids.

### EBER1 Transgenic Mice Generation, Genotyping and Cross-Breeding

*Xba*I linear fragments from pEμ^323^EBER1, pEμ^78^EBER1 and pEμ^39^EBER1 were used as transgenes for pronuclear microinjection of B6D2F2 zygotes as described previously [41]. Genotyping of founders was performed by Southern blotting as described previously [41] using a radiolabelled CR1-CR4 PCR product probe fragment. Genotyping of subsequent offspring was performed by PCR with CR8 and CR4 primers, 25 cycles, annealing at 50^o^C. Transgenic founders were bred to C57Bl/6 strain to establish lines. Transgene copy number was estimated from 1^st^ generation offspring by slot blot signal intensity by Bas-1500 PhosphorImager and MacBas V2.2 software. Transgenic mice were compared in the assays to transgene-negative sibling controls (NSC), being genetically identical (with the soul exception of the transgene) and housed together.

EμEBER1 mice were cross-bred with EμEBNA-1 [42], Eμc-myc [31] and EμN-myc mice [32]. Mice were genotyped by Southern blotting [41] and for transgenic c-myc by PCR using c-mycF and c-mycR primers (supplementary Table S2) using 12 cycles, annealing 64°C followed by 25 cycles, annealing 58°C. EμEBNA-1 and Eμc-myc mice were maintained in the C57Bl/6 strain. EμN-myc mice were maintained in the Balb/c strain, thus cross-bred sibling cohorts studied with this latter line were all F1 for the two strains and internally controlled.

### Phenotype Monitoring and Pathology

Sibling cohorts of mice were monitored for phenotype for up to 2 years. In addition, the phenotype of mice used in expression and other studies was noted. Kaplan-Meier plots were generated on the basis of lymphoma development, statistical analysis was performed using GraphPad Prism4 software using a log rank t test with p≤0.05 considered to be statistically significant. For the proportion of transgenic mice with hyperplasia and lymphoma compared to negative mice, the statistical analysis was performed using a Chi squared test. Tissues including thymus, spleen, lymph nodes and liver were fixed in 10% neutral buffered formalin, dehydrated in ethanol, embedded in paraffin wax, sectioned at 3–5 µm thickness and stained with haematoxylin and eosin for histological examination.

### RNA Extraction, RT-PCR and Quantitative RT-PCR

Total tissue RNA was extracted as described [41]. The RNA was DNase I treated (Promega) followed by acid phenol-chloroform extraction and precipitation. RNA (5 to 10µg) was used as template for the reverse-transcriptase (RT) reaction using an EBER1 gene specific reverse primer (CR4) or oligo-dT (reverse-it cDNA synthesis kit, ABgene) and MMLV RT enzyme (Invitrogen). Each reaction was performed in duplicate with or without RT enzyme. One quarter of the RT reaction was used as template for PCR using either CR8 or CR25 forward primers with CR9 reverse primer for 25 to 30 cycles annealing at 40°C. The products were separated by gel-electrophoresis, Southern blotted, probed with a radiolabelled CR1-CR4 probe and analysed by PhosphorImagery for a semi-quantitative determination. Quality of RNA was confirmed by gel electrophoresis and comparative quantities by RT-PCR with oligodT and GAPDH specific primers. For quantitative RT-PCR, the PCR reaction was conducted using the Absolute SYBR Green Rox mix kit (ABgene) according to manufacturers instructions and analysed with the DNA engine Opticon™ software (MJ Research). A standard curve was obtained using a known concentration of EBER1 plasmid DNA as template.

### Western Blotting

Proteins were extracted by polytron disruption of snap frozen samples in high salt buffer [20mM Hepes pH 7.9, 0.4M NaCl, 1mM EDTA pH 8, 1mM EGTA pH 8, 1mM DTT, 1mM PMSF, phosphatase inhibitors and protease inhibitors (Sigma)] and incubated for 10 minutes at 4°C. Following centrifugation, protein concentration was determined using the Bradford assay. Protein samples (100µg) were separated by 7.5% SDS-PAGE and western blotted as previously described [43]. Primary antibodies used were directed against the following proteins: c-Myc, N-Myc, β-tubulin, eIF2α, phospho-eIF2α (ser^51^), PKR and phospho(thr^446^)-PKR (supplementary Table S4); at 1/1000 in blocking buffer (PBST [PBS, 0.1% (v/v) Tween-20] with 5% (w/v) non-fat milk powder). Secondary goat anti-rabbit IgG horseradish peroxidase conjugated antibody was used. Detection was performed using ECL^+^ kit (GE Healthcare) following the manufacturer's instruction. Western blots for reprobing were stripped of antibody by agitation at 50°C for 1 hour in 2% SDS, 62.5 mM Tris pH 6.8, 100 mM 2 βmercaptoethanol and washed twice in PBST for 10 minutes. Densitometry of autoradiograph bands was carried out using appropriately low exposure films with the Kodak 1D software on imported images.

### Electrophoretic Mobility Shift Assay

Protein extracts were prepared as described above and assayed as previously detailed [44]. A Myc-binding E-box containing double-stranded oligonucleotide (supplementary Table S3) was end labelled with ^32^PdCTP. The probe was purified using NICK™ Columns (Amersham). For each sample, two reactions were set up: 10µg of protein extract was added to 1µg of poly dIdC (Sigma) in binding buffer [10 mM Tris, pH 7.5, 50 mM NaCl, 1 mM DTT, 1 mM EDTA and 5% glycerol]. To the duplicate, 200x of cold competitor (unlabelled oligonucleotide) was added and the samples were incubated on ice for 10 minutes. Probe (0.5 ng) was added to each sample and incubated for 20 minutes on ice. The reaction products were electrophoresed through 6% non-denaturing acrylamide gels, which were dried and exposed to film.

### Flow Cytometry and Cell Selection

Cells were isolated from spleen, thymus, peripheral and mesenteric lymph nodes (PLNs and MLNs) and bone marrow. Erythrocyte exclusion was performed with NH_4_Cl for 10 minutes to lyse the red blood cells, except for PLNs and MLNs. The cells were washed and resuspended in PBS. 10^6^ cells were stained with the antibodies indicated (supplementary Table S5) for 45 minutes at 4°C. Following two PBS washes, cells were fixed in 0.1% formaldehyde overnight before being analysed using a FACScalibur flow cytometer (Beckton Dickinson). Data were collected and analysed using CellQuest software. For B-cell/T-cell separation, cells were isolated from spleen and Peyer's patches (pooling samples from different mice as required). T-cells were first selected using Thy1.2 dynabeads (Dynal). The flow through was then used for B-cell selection with B220 dynabeads, typically achieving 90% purity in each case (assessed by flow cytometry).

### Enzyme-Linked Immunosorbant Assay (ELISA)

Serum samples were assayed at 1/20 dilution for murine interleukin-10 (IL-10) by ELISA (Biolegend #431404S) according to the manufacturer's protocol. Briefly, 96 well plates were coated with capture antibody overnight at 4°C and then blocked with assay diluent. Samples in duplicate or triplicate and standards were incubated in the wells overnight at 4°C. IL-10 was detected with a biotin-conjugated detection antibody, followed by avidin-HRP and then HRP substrate. Multiple well washes were conducted between each step. Plates were read at 450 nm. Tissue samples were assessed for IL-10 and IL-9 levels as part of a cytokine array (Raybiotech) according to manufacturer's protocol.

## Supporting Information

Figure S1 Comparative EBER1 transgene expression is shown for Peyer's patches and thymus tissues and compared to the EBV positive BL cell line Akata (AK2003) and the EBV negative derivative of this cell line Akata negative-31 (AK31). Quantitative RT-PCR was conducted using DNase treated RNA from the cell lines and tissues. The RT reaction for EBER1 was conducted using gene specific primer CR4 and for GAPDH using oligodT, using 5 µg of total RNA. An RT minus and plus reaction was performed for each sample. Q-RT-PCR was conducted using 1/4 of the sample, using primers CR8 and CR9 for EBER1 or forward and reverse primers for GAPDH (Table S2 of this supplementary information). The Q-RT-PCR data are shown in (A) and the relative expression levels (arbitrary units), normalised to GAPDH are shown for each graph (i to iv) in (B). The difference between the levels of EBER1 in the Akata cell line compared to the transgenic tissues is so great it necessitated dilution of the Akata sample by 50 fold to gain comparison. Using GAPDH as an internal control allows direct, normalised comparison between similar tissues (for example, comparing murine thymuses), but this is less accurate when comparing different tissues to one another. Between different cell types and tissues and certainly different species, it would be expected that house keeping genes (such as GAPDH) are expressed at different relative levels and as such cannot serve as normalising controls between tissues or to other species cell lines. As such, a normalised comparison of expression levels between the mouse tissues (which might be expected to contain cells not expressing EBER1) and the clonal human cell line is not possible, however, by direct comparison the amount of EBER1 cDNA in the diluted Akata sample is 100 fold higher than the highest EBER1 thymus sample (that of line 142).
(0.23 MB PPT)

Figure S2 Transgene expression in non-lymphoid tissues was assessed in lines 136, 127, 131 and 137 from DNaseI-treated, total RNA derived from tissues of 2 to 4 month old mice by RT-PCR using a gene specific RT primer. PCR products were Southern blotted and hybridised with an EBER1 probe. +indicates inclusion of RT, - indicates no RT added and is indicative of signal from any residual DNA in the sample. PCR controls: negative (N) water only, positive (P) amplified from plasmid DNA. Tissues include: stomach, lung, heart, small intestine (S.int), testis, ovaries, uterus, kidney (kid), oesophagus (oesp), trachea, brain, tongue, salivary gland (SG), nasopharyngeal region (NPR), muscle and ears. Note: to detect very low levels of expression, exposure times were maximised, such that in some cases, a low signal (presumably from tiny amounts of contaminating DNA) can be detected in RT- samples. Thus where RT- and RT+ samples show a band of similar intensity, this would imply no detectable expression in that tissue.
(1.49 MB PPT)

Figure S3 Flow cytometric analysis of surface markers of pre-phenotypic lymphoid tissues of EμEBER1 transgenic mice. The lymphoid tissues (spleen, thymus, peripheral and mesenteric lymph nodes and bone marrow) were examined by flow cytometry from young mice (2–4 months old) of the lines 127 (shown) and 131 (not shown), prior to the development of phenotype. No difference between transgenic and NSC tissues were found for fluorochrome-conjugated antibody staining against B220, CD5, CD23, CD43, IgM, IgG, IgA, CD3, Thy1.2, CD2, CD4 and CD8, except for B220/CD5 staining of Peyer's patch cells of mice of line 127 as shown in the representative examples described. First panel a - c: Spleen, bone marrow and Peyer's patches were collected from three line 127 mice (right) and 3 NSC (left) and each pooled. 106 cells were stained with anti-B220/FITC, CD3/PE (a), B220/FITC, Thy1.2/PE (b) and B220/FITC, CD3/PE (c), all showing no difference between transgenic and NSC. The percentage of cells in each quadrant is indicated. Second panel d - f: Peyer's patches stained with anti-CD5/FITC, CD3/PE (d), CD5/FITC, B220/PE (e) are shown. The forward (FSC) and side scatter (SSC) for the CD5/FITC, B220/PE stain is shown in f. The percentage of cells in each quadrant is indicated. While no difference was observed in the CD5+/CD3+ T-cell population (d), the CD5+ B-cell population is largely B220low in the NSC samples while it is B220neg in the transgenic sample (e), suggesting the B1a population might be increased in this tissue in the transgenic line. This is supported by the increase in large, granular cells (f). Repetition of the experiment with further mice gave the same result.
(0.12 MB PPT)

Figure S4 Immunoglubulin heavy chain gene (IgH) rearrangement in the EBER1 tumour samples were assessed by Southern blotting of EcoRI digested genomic DNA. DNA derived from tumour tissues of an EμN-myc mouse (positive control), two EμEBER1 line 127 mice (127.49 and 127.37) and NSC mouse were examined. The membrane was hybridised with an IgH J-region sequence probe showing rearrangements (circles to left of track) compared to the endogenous germ line band (E). S = spleen, MLN = mesenteric lymph node. The clonal IgH rearrangements detected in the EBER tumour samples, like that of the N-myc tumour sample suggests that the tumours are of B-cell origin, supporting the flow cytometry data.
(0.15 MB PPT)

Table S1 Numbers of founder mice developed with the three EBER1 construct variants are shown with the number of transgenic lines successfully generated from these. Of the eleven lines tested for expression, ten showed expression while one (line Eμ39EBER1.133) showed no expression in any tissue tested. The ID of the expressing lines is given with the integrated transgene copy number indicated in parentheses.
(0.03 MB PPT)

Table S2 Oligonucleotide sequences used for PCR.
(0.05 MB PDF)

Table S3 Oligonucleotide sequences used for EMSA.
(0.07 MB PDF)

Table S4 Antibodies used in western analyses.
(0.05 MB PPT)

Table S5 Antibodies used for flow cytometry.
(0.03 MB PPT)
