# Supplementary material for: Lymphoid Hyperplasia and Lymphoma in Transgenic Mice Expressing the Small Non-Coding RNA, EBER1 of Epstein-Barr Virus
Source: PLoS One. 2010 Feb 8;5(2):e9092. doi: 10.1371/journal.pone.0009092 (PMC2817001; doi:10.1371/journal.pone.0009092)
Supplement: Figure S1 — Comparative EBER1 transgene expression is shown for Peyer's patches and thymus tissues and compared to the EBV positive BL cell line Akata (AK2003) and the EBV negative derivative of this cell line Akata negative-31 (AK31). Quantitative RT-PCR was conducted using DNase treated RNA from the cell lines and tissues. The RT reaction for EBER1 was conducted using gene specific primer CR4 and for GAPDH using oligodT, using 5 µg of total RNA. An RT minus and plus reaction was performed for each sample. Q-RT-PCR was conducted using 1/4 of the sample, using primers CR8 and CR9 for EBER1 or forward and reverse primers for GAPDH (Table S2 of this supplementary information). The Q-RT-PCR data are shown in (A) and the relative expression levels (arbitrary units), normalised to GAPDH are shown for each graph (i to iv) in (B). The difference between the levels of EBER1 in the Akata cell line compared to the transgenic tissues is so great it necessitated dilution of the Akata sample by 50 fold to gain comparison. Using GAPDH as an internal control allows direct, normalised comparison between similar tissues (for example, comparing murine thymuses), but this is less accurate when comparing different tissues to one another. Between different cell types and tissues and certainly different species, it would be expected that house keeping genes (such as GAPDH) are expressed at different relative levels and as such cannot serve as normalising controls between tissues or to other species cell lines. As such, a normalised comparison of expression levels between the mouse tissues (which might be expected to contain cells not expressing EBER1) and the clonal human cell line is not possible, however, by direct comparison the amount of EBER1 cDNA in the diluted Akata sample is 100 fold higher than the highest EBER1 thymus sample (that of line 142). (0.23 MB PPT) [file pone.0009092.s001.ppt]

## Slide 1
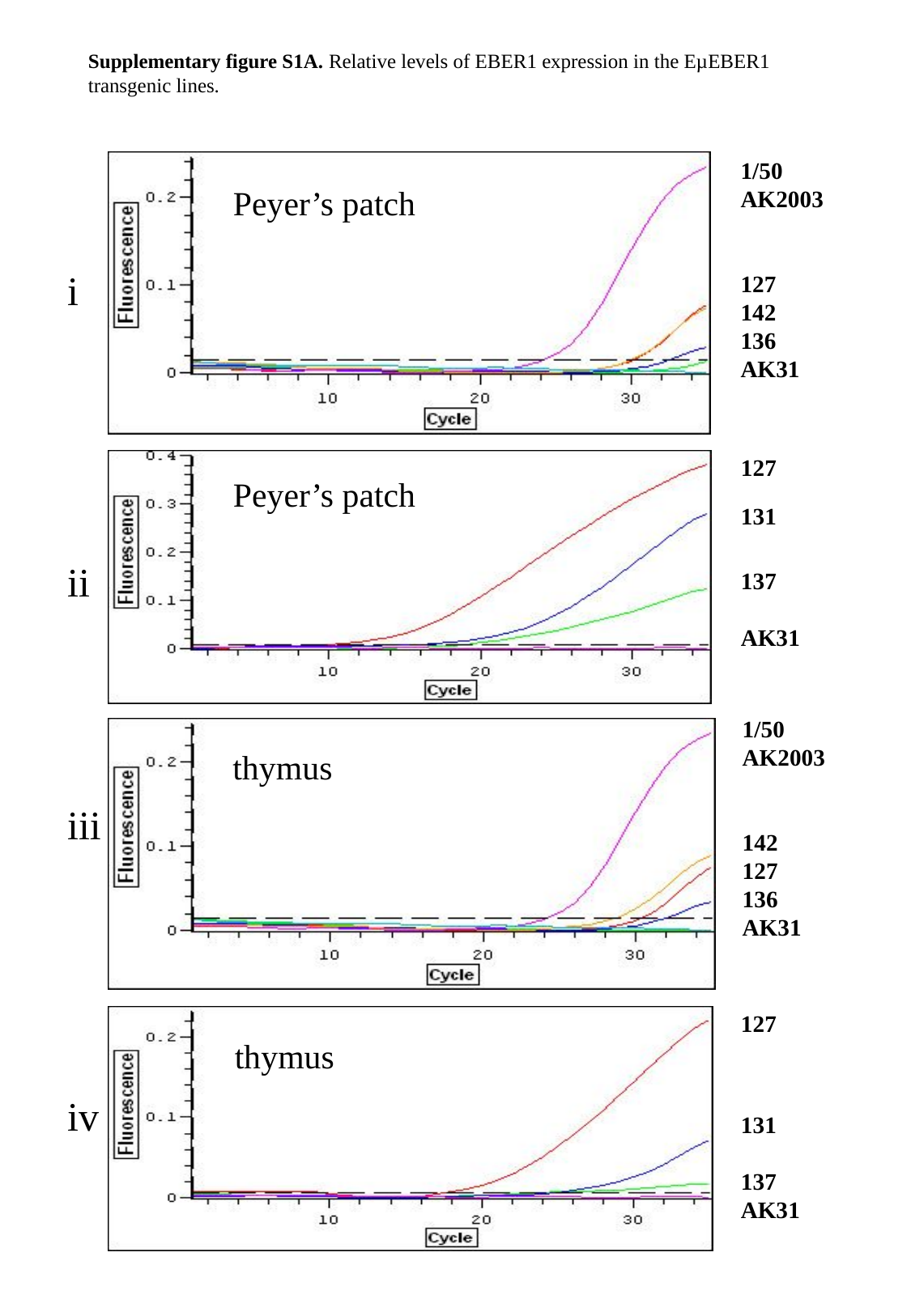

Supplementary figure S1A. Relative levels of EBER1 expression in the EµEBER1 transgenic lines.
1/50
AK2003
127
142
136
AK31
Peyer’s patch
i
ii
iii
iv
127
131
137
AK31
Peyer’s patch
1/50
AK2003
142
127
136
AK31
thymus
127
131
137
AK31
thymus

## Slide 2
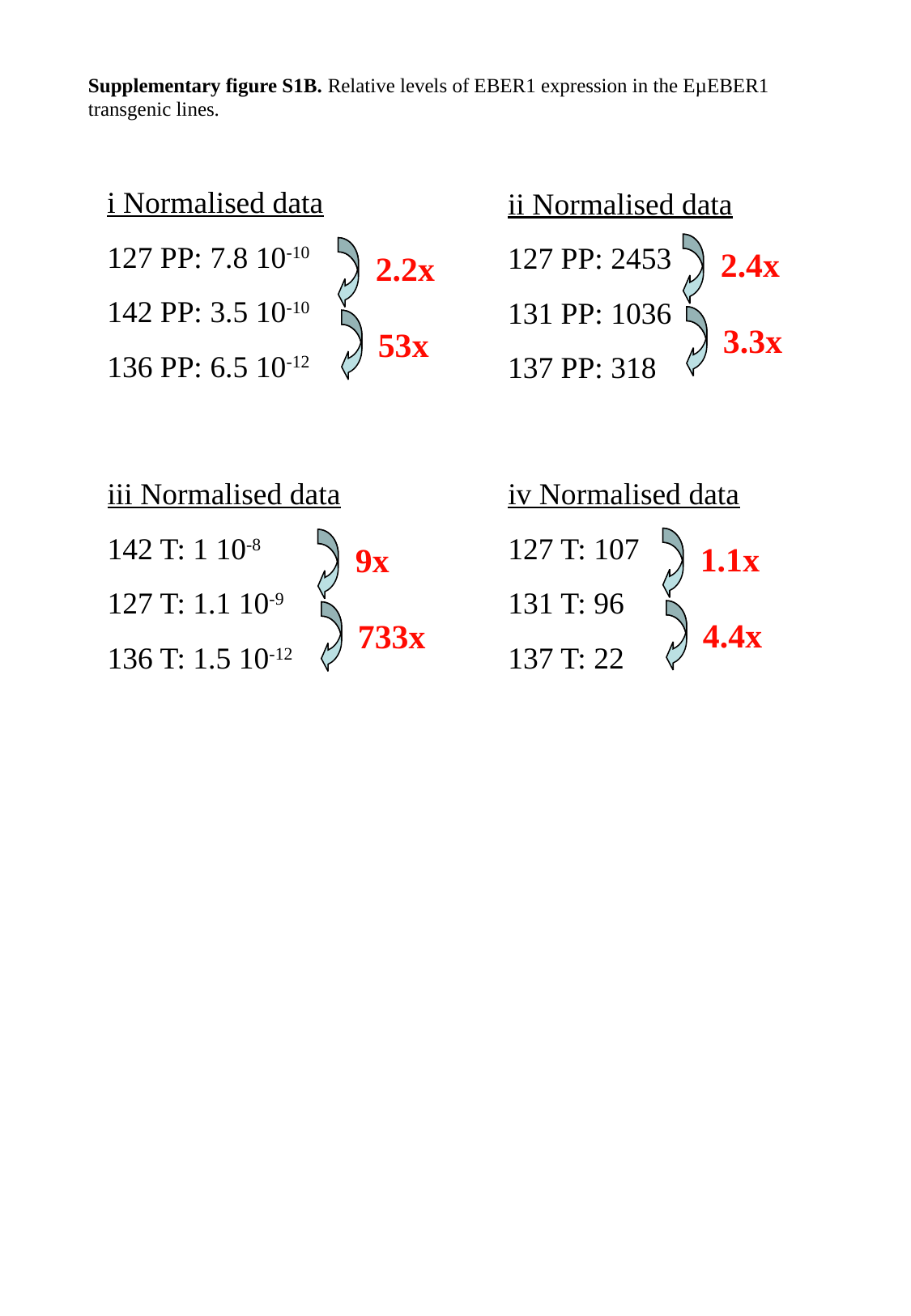

Supplementary figure S1B. Relative levels of EBER1 expression in the EµEBER1 transgenic lines.
i Normalised data
127 PP: 7.8 10-10
142 PP: 3.5 10-10
136 PP: 6.5 10-12
2.2x
53x
ii Normalised data
127 PP: 2453
131 PP: 1036
137 PP: 318
2.4x
3.3x
iii Normalised data
142 T: 1 10-8
127 T: 1.1 10-9
136 T: 1.5 10-12
9x
733x
iv Normalised data
127 T: 107
131 T: 96
137 T: 22
1.1x
4.4x
