# Supplementary material for: Lymphoid Hyperplasia and Lymphoma in Transgenic Mice Expressing the Small Non-Coding RNA, EBER1 of Epstein-Barr Virus
Source: PLoS One. 2010 Feb 8;5(2):e9092. doi: 10.1371/journal.pone.0009092 (PMC2817001; doi:10.1371/journal.pone.0009092)
Supplement: Figure S2 — Transgene expression in non-lymphoid tissues was assessed in lines 136, 127, 131 and 137 from DNaseI-treated, total RNA derived from tissues of 2 to 4 month old mice by RT-PCR using a gene specific RT primer. PCR products were Southern blotted and hybridised with an EBER1 probe. +indicates inclusion of RT, - indicates no RT added and is indicative of signal from any residual DNA in the sample. PCR controls: negative (N) water only, positive (P) amplified from plasmid DNA. Tissues include: stomach, lung, heart, small intestine (S.int), testis, ovaries, uterus, kidney (kid), oesophagus (oesp), trachea, brain, tongue, salivary gland (SG), nasopharyngeal region (NPR), muscle and ears. Note: to detect very low levels of expression, exposure times were maximised, such that in some cases, a low signal (presumably from tiny amounts of contaminating DNA) can be detected in RT- samples. Thus where RT- and RT+ samples show a band of similar intensity, this would imply no detectable expression in that tissue. (1.49 MB PPT) [file pone.0009092.s002.ppt]

## Slide 1
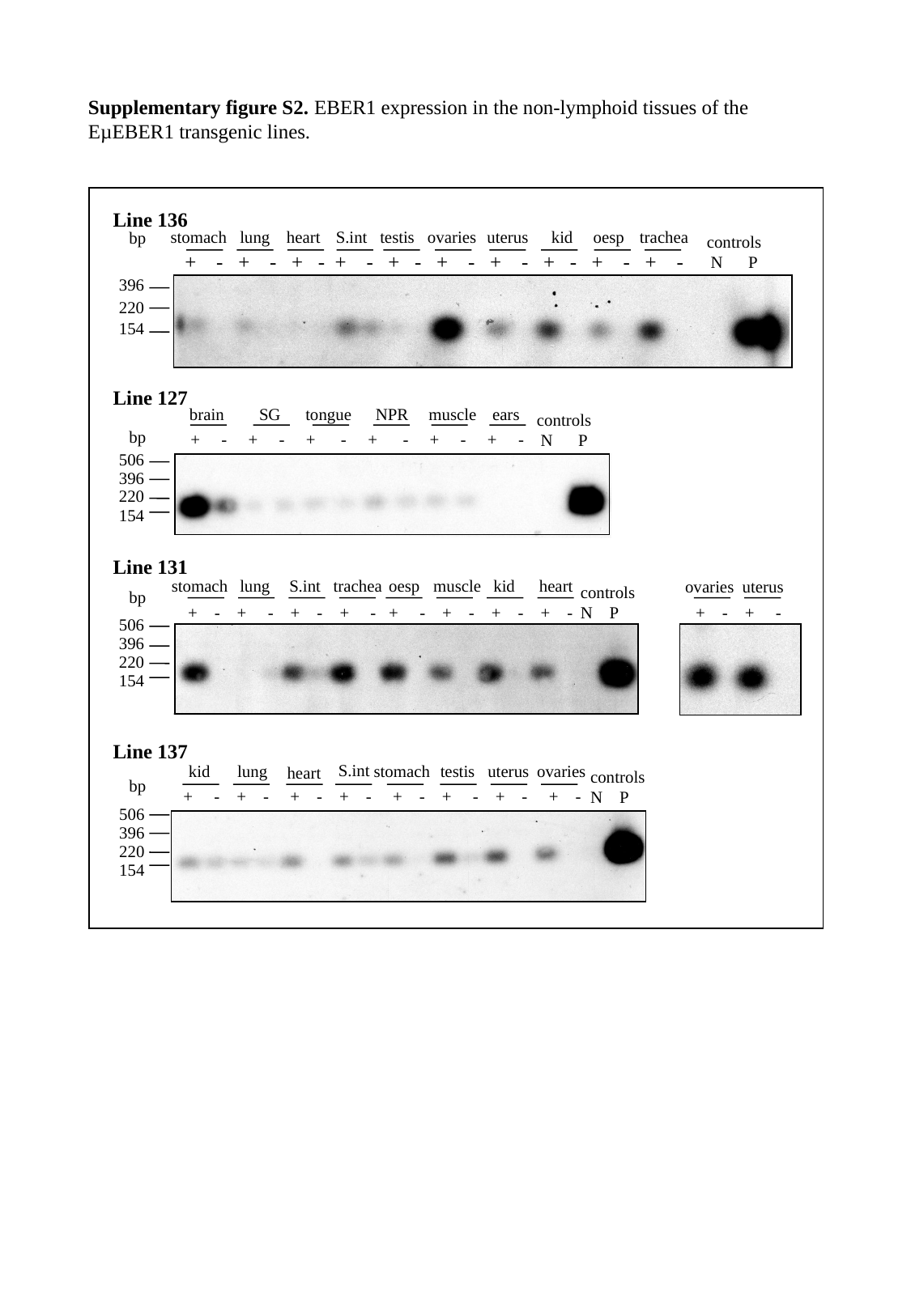

Supplementary figure S2. EBER1 expression in the non-lymphoid tissues of the EµEBER1 transgenic lines.
Line 136
stomach
lung
heart
S.int
ovaries
uterus
oesp
trachea
testis
kid
bp
controls
N P
+ - + - + - + - + - + - + - + - + - + -
396
220
154
Line 127
SG
tongue
muscle
NPR
brain
ears
controls
N P
bp
 + - + - + - + - + - + -
506
396
220
154
Line 131
S.int
oesp
stomach
lung
trachea
muscle
kid
heart
ovaries
uterus
controls
N P
bp
+ - + - + - + - + - + - + - + -
+ - + -
506
396
220
154
Line 137
S.int
kid
lung
stomach
testis
uterus
ovaries
heart
controls
N P
bp
+ - + - + - + - + - + - + - + -
506
396
220
154
