# Supplementary material for: Lymphoid Hyperplasia and Lymphoma in Transgenic Mice Expressing the Small Non-Coding RNA, EBER1 of Epstein-Barr Virus
Source: PLoS One. 2010 Feb 8;5(2):e9092. doi: 10.1371/journal.pone.0009092 (PMC2817001; doi:10.1371/journal.pone.0009092)
Supplement: Figure S3 — Flow cytometric analysis of surface markers of pre-phenotypic lymphoid tissues of EμEBER1 transgenic mice. The lymphoid tissues (spleen, thymus, peripheral and mesenteric lymph nodes and bone marrow) were examined by flow cytometry from young mice (2–4 months old) of the lines 127 (shown) and 131 (not shown), prior to the development of phenotype. No difference between transgenic and NSC tissues were found for fluorochrome-conjugated antibody staining against B220, CD5, CD23, CD43, IgM, IgG, IgA, CD3, Thy1.2, CD2, CD4 and CD8, except for B220/CD5 staining of Peyer's patch cells of mice of line 127 as shown in the representative examples described. First panel a - c: Spleen, bone marrow and Peyer's patches were collected from three line 127 mice (right) and 3 NSC (left) and each pooled. 106 cells were stained with anti-B220/FITC, CD3/PE (a), B220/FITC, Thy1.2/PE (b) and B220/FITC, CD3/PE (c), all showing no difference between transgenic and NSC. The percentage of cells in each quadrant is indicated. Second panel d - f: Peyer's patches stained with anti-CD5/FITC, CD3/PE (d), CD5/FITC, B220/PE (e) are shown. The forward (FSC) and side scatter (SSC) for the CD5/FITC, B220/PE stain is shown in f. The percentage of cells in each quadrant is indicated. While no difference was observed in the CD5+/CD3+ T-cell population (d), the CD5+ B-cell population is largely B220low in the NSC samples while it is B220neg in the transgenic sample (e), suggesting the B1a population might be increased in this tissue in the transgenic line. This is supported by the increase in large, granular cells (f). Repetition of the experiment with further mice gave the same result. (0.12 MB PPT) [file pone.0009092.s003.ppt]

## Slide 1
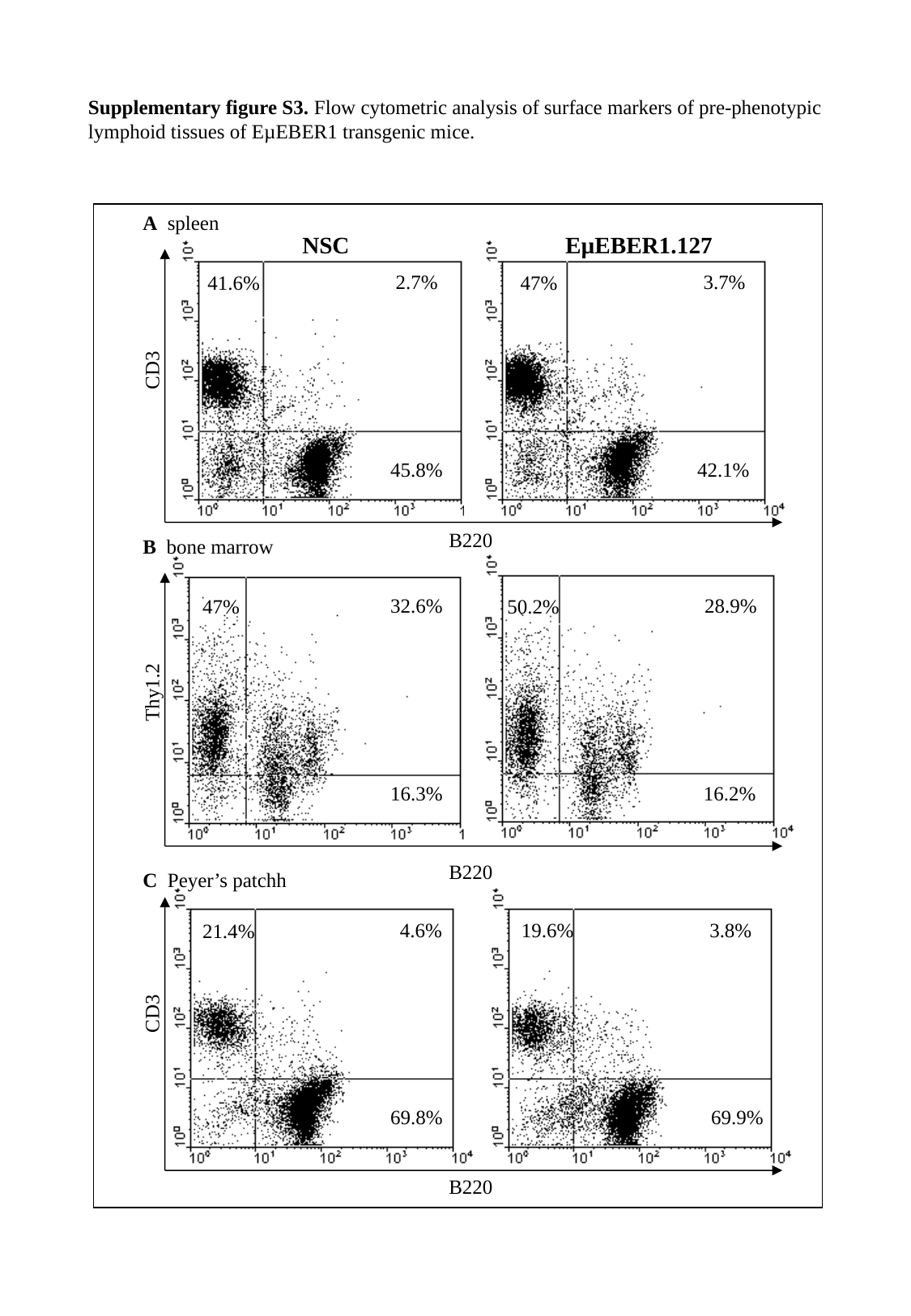

Supplementary figure S3. Flow cytometric analysis of surface markers of pre-phenotypic lymphoid tissues of EµEBER1 transgenic mice.
A spleen
 NSC
EµEBER1.127
2.7%
3.7%
47%
41.6%
CD3
45.8%
42.1%
B220
B bone marrow
32.6%
28.9%
50.2%
47%
Thy1.2
16.3%
16.2%
B220
C Peyer’s patchh
4.6%
3.8%
19.6%
21.4%
CD3
69.8%
69.9%
B220

## Slide 2
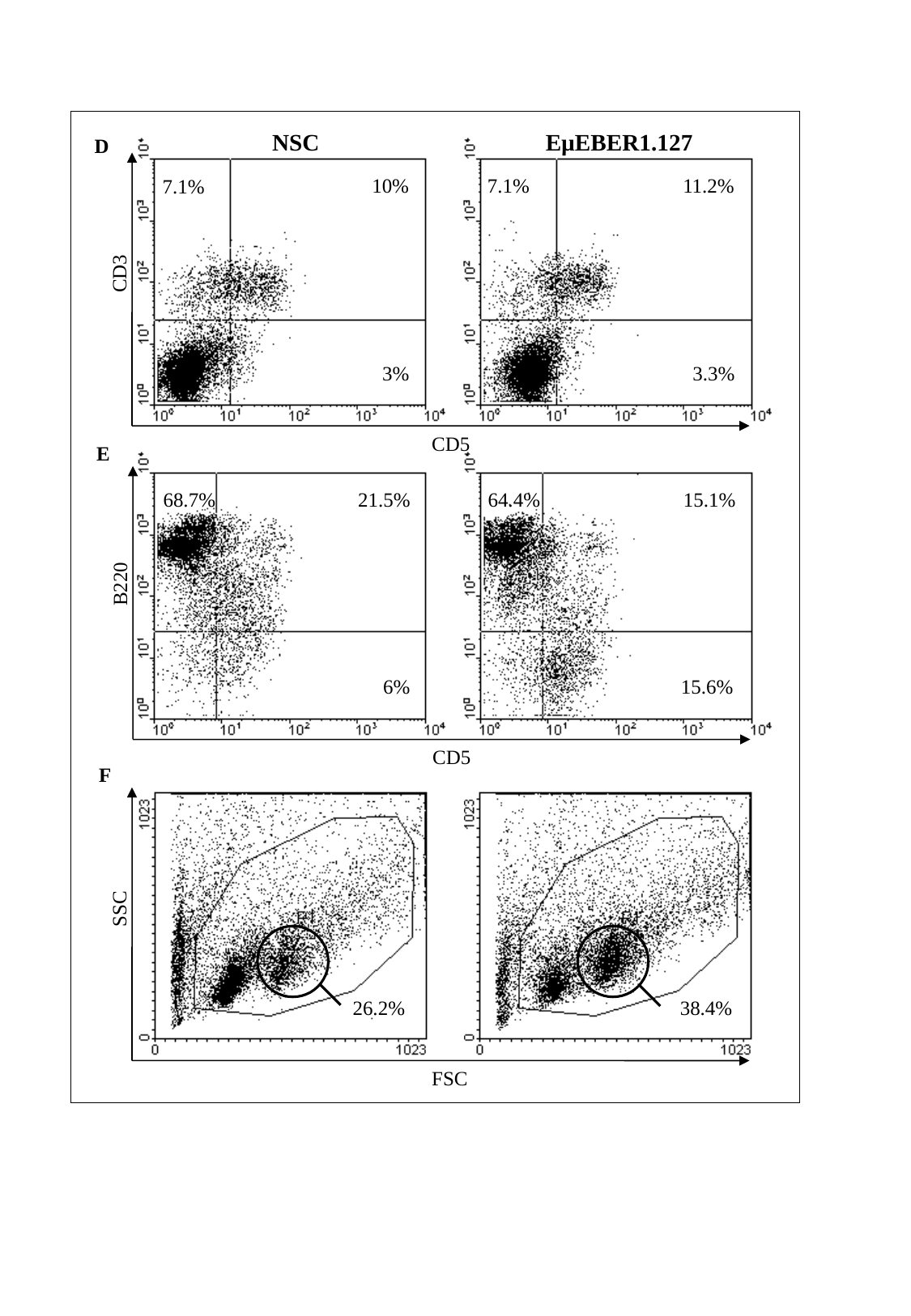

NSC
EµEBER1.127
D
10%
11.2%
7.1%
7.1%
CD3
3%
3.3%
CD5
E
21.5%
15.1%
64.4%
68.7%
B220
6%
15.6%
CD5
F
SSC
26.2%
38.4%
FSC
