# Supplementary material for: Lymphoid Hyperplasia and Lymphoma in Transgenic Mice Expressing the Small Non-Coding RNA, EBER1 of Epstein-Barr Virus
Source: PLoS One. 2010 Feb 8;5(2):e9092. doi: 10.1371/journal.pone.0009092 (PMC2817001; doi:10.1371/journal.pone.0009092)
Supplement: Figure S4 — Immunoglubulin heavy chain gene (IgH) rearrangement in the EBER1 tumour samples were assessed by Southern blotting of EcoRI digested genomic DNA. DNA derived from tumour tissues of an EμN-myc mouse (positive control), two EμEBER1 line 127 mice (127.49 and 127.37) and NSC mouse were examined. The membrane was hybridised with an IgH J-region sequence probe showing rearrangements (circles to left of track) compared to the endogenous germ line band (E). S = spleen, MLN = mesenteric lymph node. The clonal IgH rearrangements detected in the EBER tumour samples, like that of the N-myc tumour sample suggests that the tumours are of B-cell origin, supporting the flow cytometry data. (0.15 MB PPT) [file pone.0009092.s004.ppt]

## Slide 1
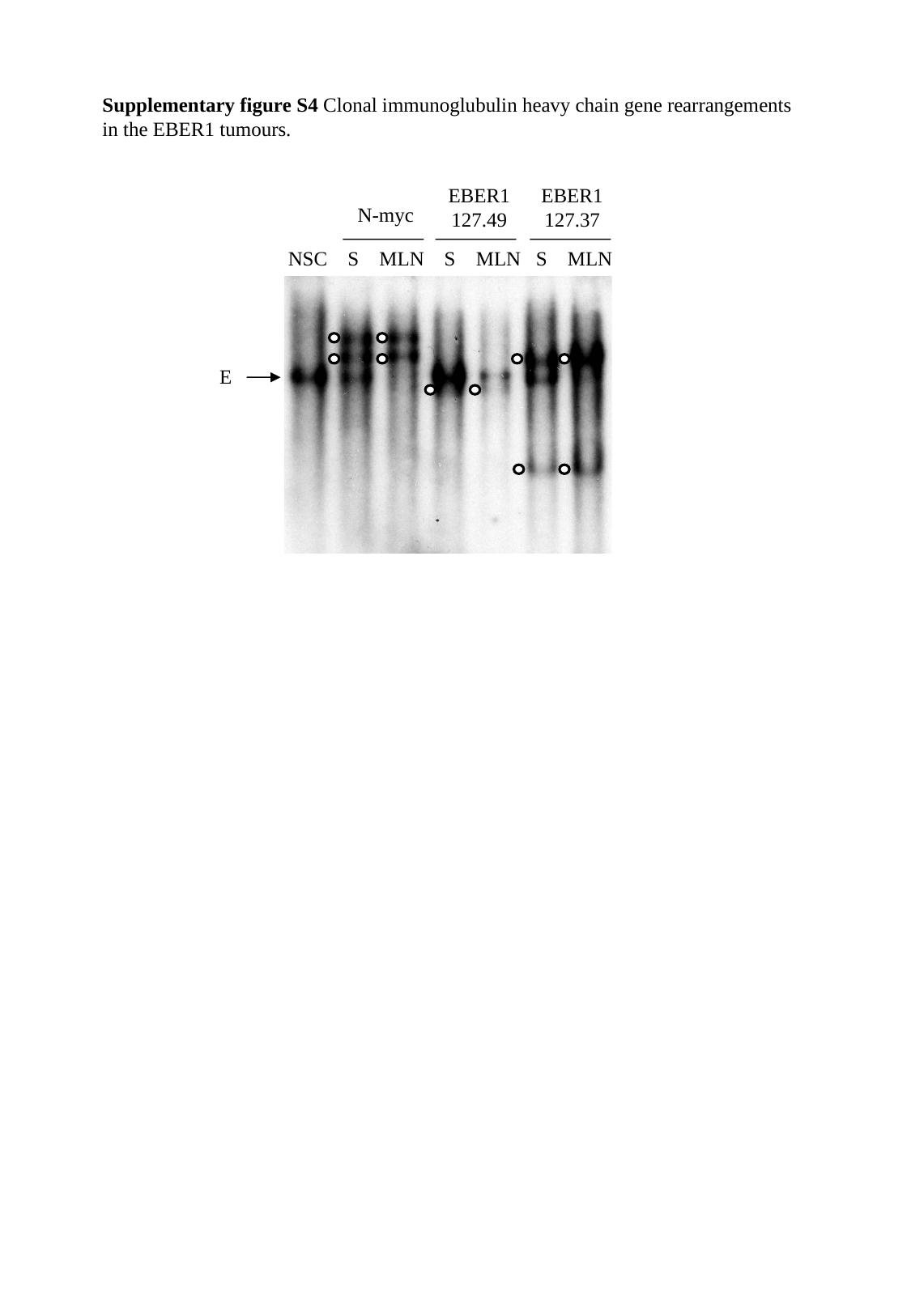

Supplementary figure S4 Clonal immunoglubulin heavy chain gene rearrangements in the EBER1 tumours.
EBER1
127.49
EBER1
127.37
N-myc
NSC S MLN S MLN S MLN
E
