# Supplementary figures and images for: Lymphoid Hyperplasia and Lymphoma in Transgenic Mice Expressing the Small Non-Coding RNA, EBER1 of Epstein-Barr Virus
Source: PLoS One. 2010 Feb 8;5(2):e9092. doi: 10.1371/journal.pone.0009092 (PMC2817001; doi:10.1371/journal.pone.0009092)

## Slide 1
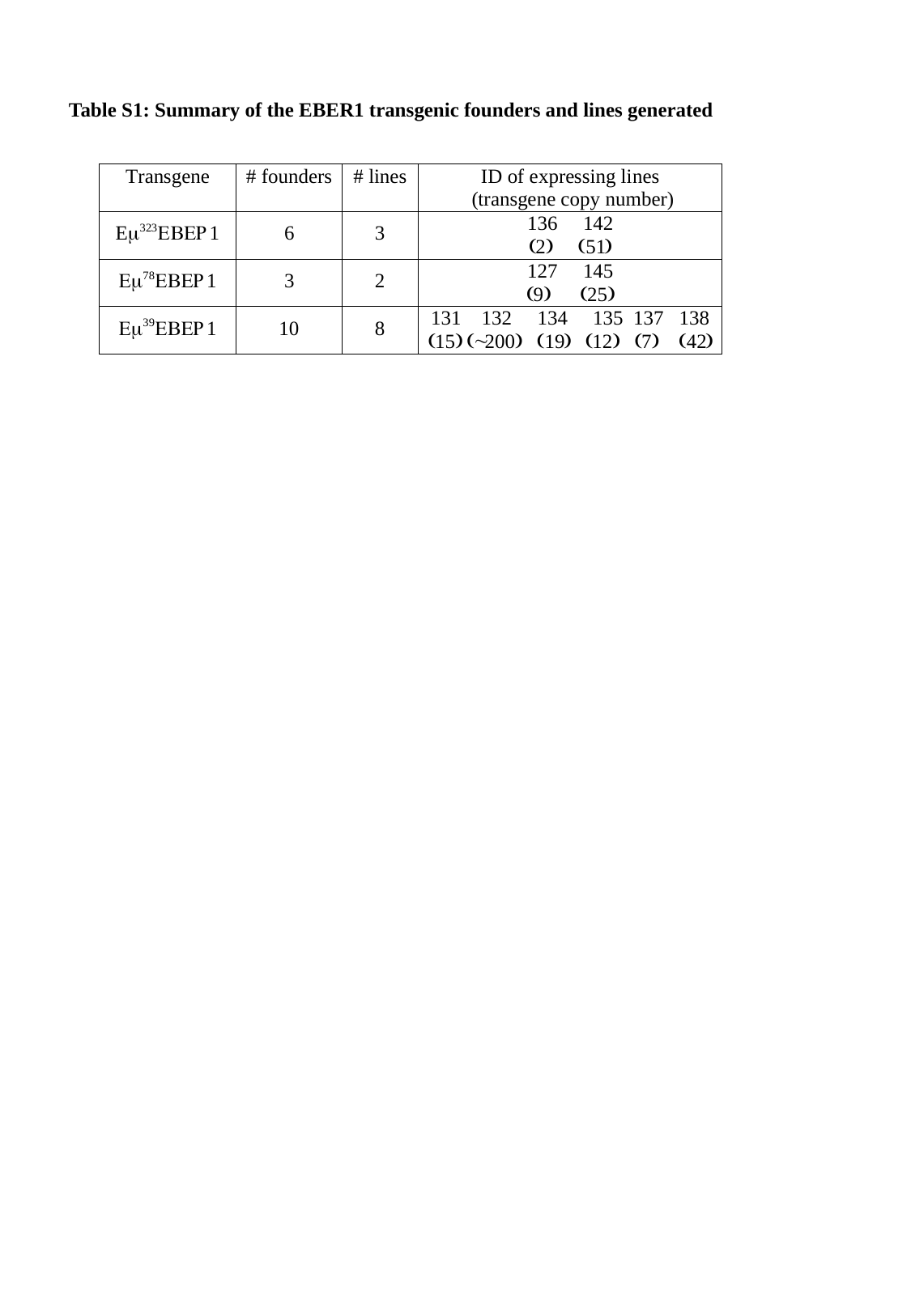

Table S1: Summary of the EBER1 transgenic founders and lines generated

Supplement: Table S1 — Numbers of founder mice developed with the three EBER1 construct variants are shown with the number of transgenic lines successfully generated from these. Of the eleven lines tested for expression, ten showed expression while one (line Eμ39EBER1.133) showed no expression in any tissue tested. The ID of the expressing lines is given with the integrated transgene copy number indicated in parentheses. (0.03 MB PPT) [file pone.0009092.s005.ppt]

## Slide 1
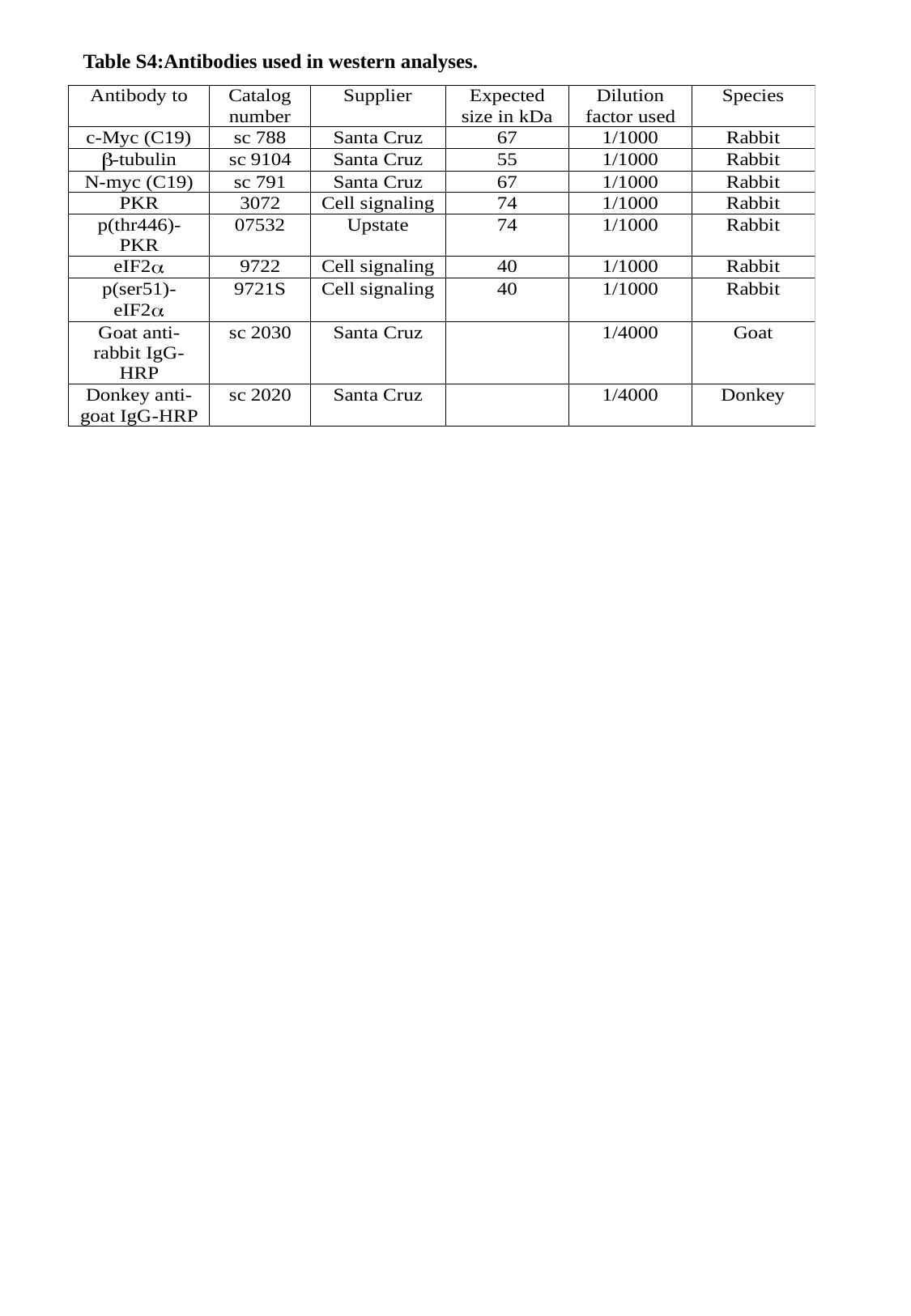

Table S4:Antibodies used in western analyses.

Supplement: Table S4 — Antibodies used in western analyses. (0.05 MB PPT) [file pone.0009092.s008.ppt]

## Slide 1
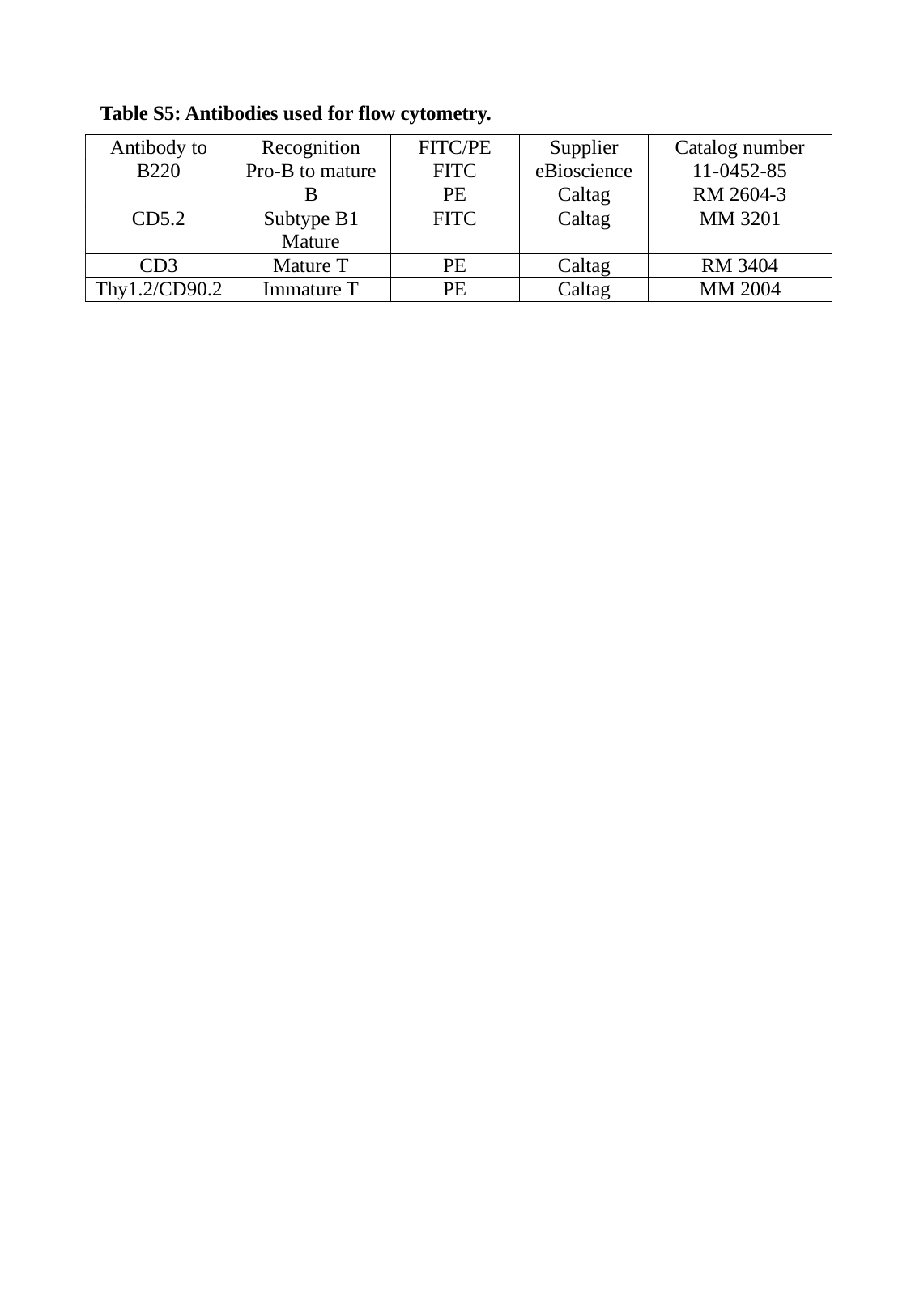

Table S5: Antibodies used for flow cytometry.

Supplement: Table S5 — Antibodies used for flow cytometry. (0.03 MB PPT) [file pone.0009092.s009.ppt]
