# Supplementary material for: Lymphoid Hyperplasia and Lymphoma in Transgenic Mice Expressing the Small Non-Coding RNA, EBER1 of Epstein-Barr Virus
Source: PLoS One. 2010 Feb 8;5(2):e9092. doi: 10.1371/journal.pone.0009092 (PMC2817001; doi:10.1371/journal.pone.0009092)
Supplement: Table S2 — Oligonucleotide sequences used for PCR. (0.05 MB PDF) [file pone.0009092.s006.pdf]

Supplementary materials

Table S2: Oligonucleotide sequences used for PCR.

Primer name	5´ to 3´ primer sequence
CR1	gtgtgtgtgaattcgtcagcctgcaaggtg
CR2	gtgtgtgtgaattcactatagcaaaccccg
CR3	gtgtgtgtgaattctcttgaggagatgtag
CR4	gtgtgtgtctcgagaaaacatgcggaccac
CR8	aggacctacgctgc
CR9	tacttgaccgaagac
CR25	gtagacactgcaaaacctc
GAPDH F	tccaccaccctgttgctgta
GAPDH R	accacagtccatgccatcac
c-mycF	cagctggcgtaatagcgaagag
c-mycR	ctgtgactggtgagtactcaacc
