# Supplementary material for: Lymphoid Hyperplasia and Lymphoma in Transgenic Mice Expressing the Small Non-Coding RNA, EBER1 of Epstein-Barr Virus
Source: PLoS One. 2010 Feb 8;5(2):e9092. doi: 10.1371/journal.pone.0009092 (PMC2817001; doi:10.1371/journal.pone.0009092)
Supplement: Table S3 — Oligonucleotide sequences used for EMSA. (0.07 MB PDF) [file pone.0009092.s007.pdf]

Table 3: Oligonucleotide sequences used for EMSA.

Primer name	5′ to 3′ primer sequence
EMSAMycF	gagcggaagcagac**cacgtg**gtctgcttcc
EMSAMycR	gtagggaagcagaccacgtggtctgcttcc

The E-box Myc binding consensus motif is shown in bold in the forward primer.
